# Author Correction: Disentangling the architectural and non-architectural functions of CTCF and cohesin in gene regulation

**DOI:** 10.1038/s41588-025-02477-8

**Published:** 2025-12-16

**Authors:** Takeo Narita, Sinan Kilic, Yoshiki Higashijima, Natalie M. Scherer, Georgios Pappas, Elina Maskey, Chunaram Choudhary

**Affiliations:** 1 Proteomic Program, The Novo Nordisk Foundation Center for Protein Research, Department of Cellular and Molecular Medicine, Faculty of Health and Medical Sciences, University of Copenhagen, Copenhagen, Denmark; 2 Institute for Promotion of Tenure Track, University of Miyazaki, Miyazaki, Japan

**Keywords:** Gene regulation, Epigenetics

Correction to: *Nature Genetics* 10.1038/s41588-025-02404-x, published online 18 November 2025.

In the version of this article initially published, the criteria for defining statistically significantly regulated genes erroneously omitted to mention fold-change threshold applied. To reflect this, the text ”A-485 significantly (*P*_adj_ < 0.05) downregulated >1,000 genes, whereas RAD21^AID^ depletion altered only one (Extended Data Fig. 1b)” should read “A-485 significantly (*P*_adj_ < 0.05, ≥2-fold change) downregulated >1,000 genes, whereas RAD21^AID^ depletion altered only one (Extended Data Fig. 1b).”

The authors unwittingly omitted a reference to a paper that suggested that CTCF may function as a transcription activator. This paper is now cited as ref. 73 (Zhang, H. et al. CTCF and transcription influence chromatin structure re-configuration after mitosis. *Nat Commun*. **12**, 5157 (2021)). To cite this, the following sentence was inserted in the middle of the fifth paragraph in the Discussion: “Some recent studies suggested that promoter-proximal CTCF binding facilitates interaction with distal enhancers^15,16^, while others hinted that CTCF may also act as a transcriptional activator^26,66,73^. However, they did not directly demonstrate this activator role or separate it from CTCF’s architectural functions.”

Additionally, an error was introduced by the Publisher in the first paragraph of the Results, where the text “Earlier studies assessed transcription changes after acute RAD21 or CTCF depletion…” originally cited refs. 20,25–44 but should have cited refs. 20, 25, 26.

These changes do not change the results or the conclusions of the article. The changes have been made in the HTML and PDF versions of the article.

