# Supplementary material for: Disentangling the architectural and non-architectural functions of CTCF and cohesin in gene regulation
Source: Nat Genet. 2025 Nov 18;57(12):3137–51. doi: 10.1038/s41588-025-02404-x (PMC12695661; doi:10.1038/s41588-025-02404-x)
Supplement: Supplementary file 1 — Supplementary Figs. 1–4, Tables 1 and 2, Notes 1–4, Methods and References. [file 41588_2025_2404_MOESM1_ESM.pdf]

nature genetics

Supplementary information https://doi.org/10.1038/s41588-025-02404-x

Disentangling the architectural and non-architectural functions of CTCF and cohesin in gene regulation

In the format provided by the authors and unedited

Table of Contents

Supplementary Figures 1-4
Supplementary Tables 1-2
Supplementary Notes 1-4
Supplementary Methods
Supplementary References

Supplementary Figures

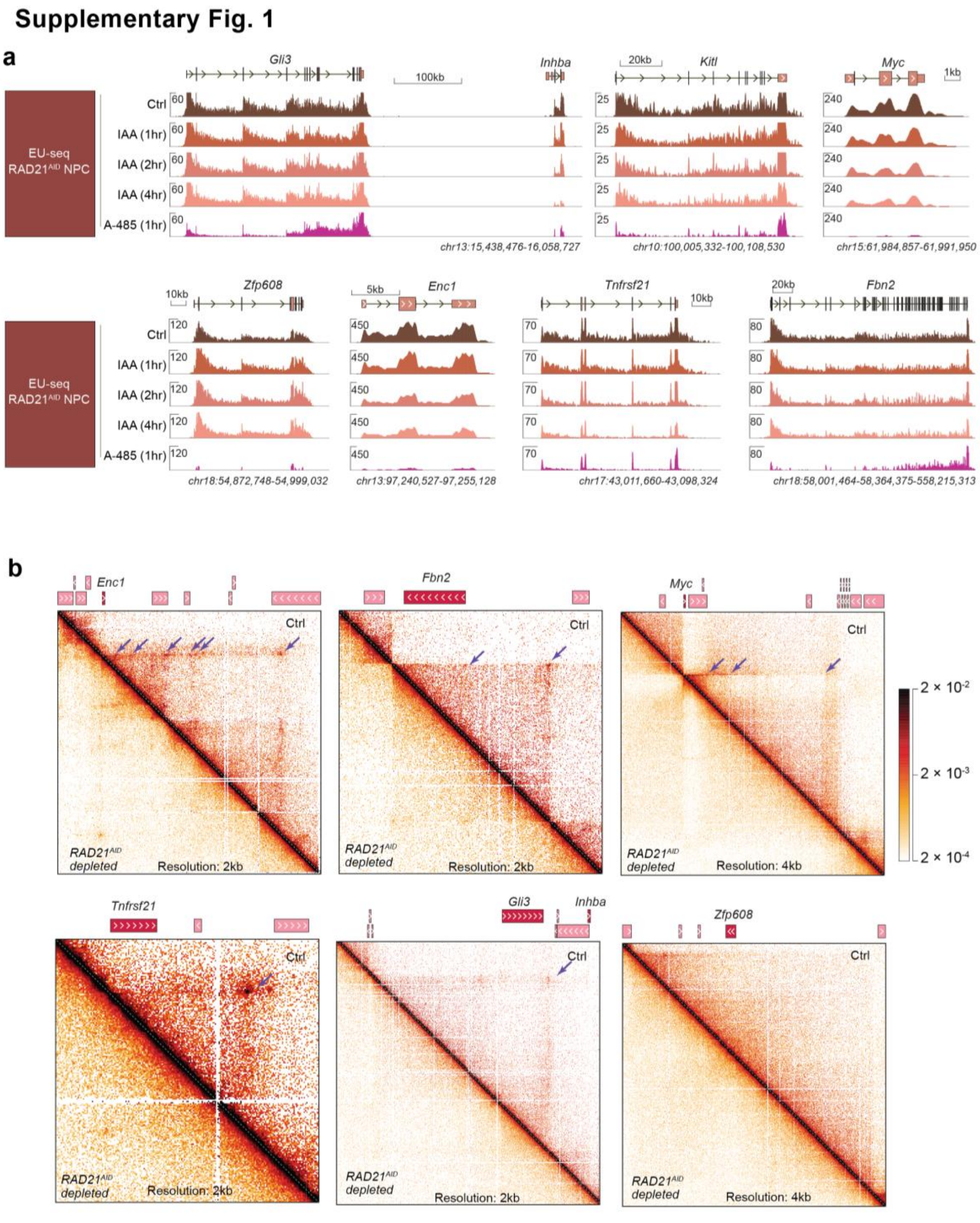

Supplementary Fig. 1. RAD21^{AID} depletion causes gene-selective transcription downregulation in NPC.

a, Genome browser tracks showing regulation of known (*Fbn2*, *Myc, Gli3*, *Zfp608*) and putative (*Inhba*, *Tnfrsf21*) enhancer targets after RAD21^{AID} depletion and A-485 treatment in NPC. Some of these targets (*Fbn2*, *Myc, Enc1, Tnfrsf21*) are downregulated both by RAD21^{AID} depletion and A-485 treatment, but others (*Gli3, Zfp608*) are only downregulated by A-485. Of note, *Gli3* expression is only decreased after A-485 treatment, but the *Gli3* proximal gene, *Inhba*, is downregulated by both RAD21^{AID} depletion and A-485 treatment.

b, Micro-C detected chromatin interactions in the indicated loci in wild-type mESC, and a change in loop strength after RAD21^{AID} depletion. Micro-C data are from reference[1]. Putative chromatin loops (marked by

arrows) are detectable in mESC for some of the genes regulated by RAD21^{AID} depletion in NPC (*Enc*1, *Fbn2*, *Myc, Gli3*, *Tnfrsf21*), but in others they are not visible (*Zfp608*).

Supplementary Fig. 2

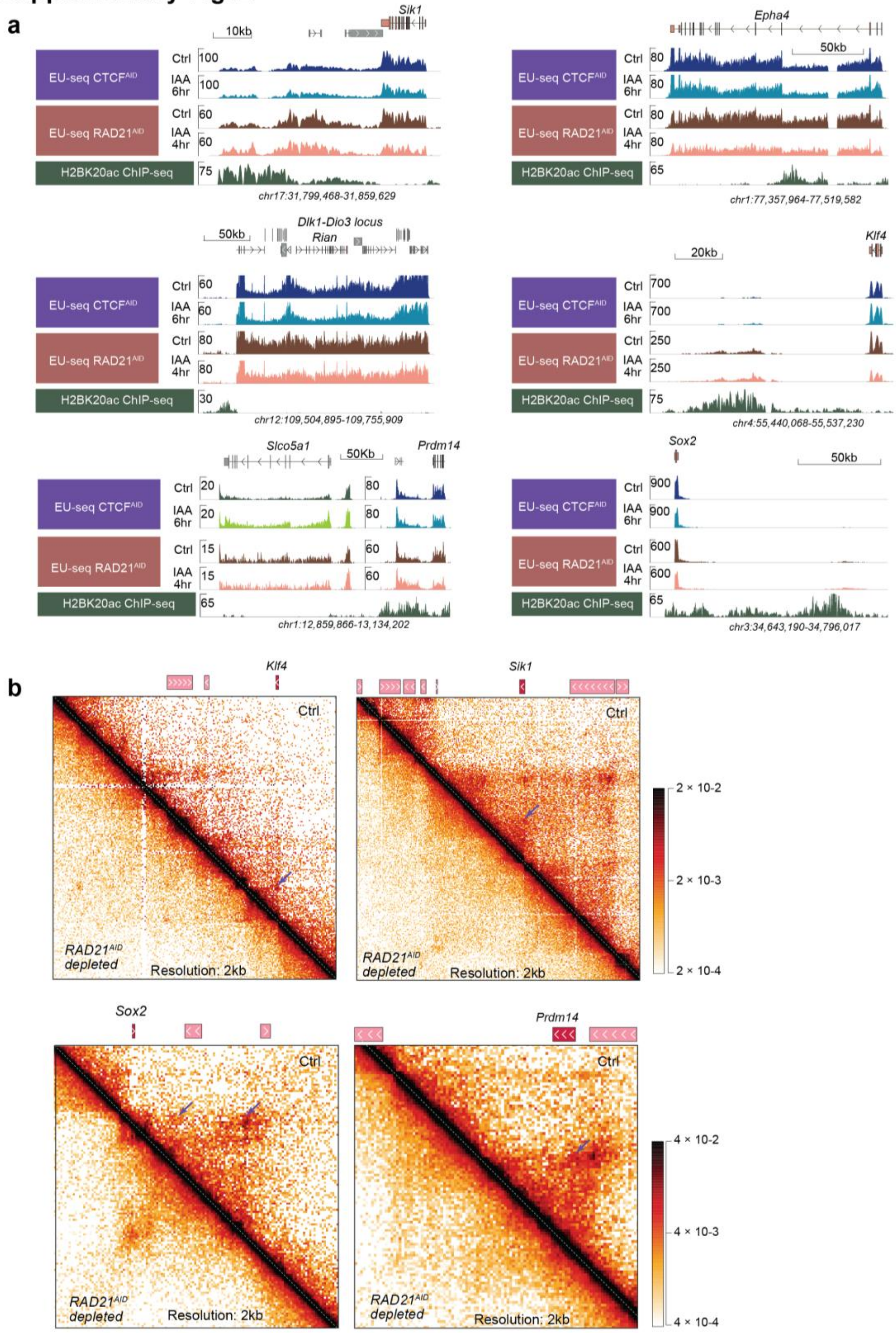

Supplementary Fig. 2. RAD21^{AID} and CTCF^{AID} depletion decrease the expression of known enhancer targets, and RAD21^{AID} depletion causes loss of chromatin loops in the regulated gene loci.

a, Genome browser tracks showing downregulation of known enhancer target genes by RAD21^{AID} and CTCF^{AID} depletion in mESC. H2BK20ac ChIP tracks show the position of known enhancers for the indicated genes. Of note, *Sik1*, *Klf4*, and *Sox2* show variable levels of nascent transcription in non-genic regions marked by H2BK20a, but this transcription in non-genic regions is not reduced by RAD21^{AID} and CTCF^{AID} depletion, whereas transcription of the known target genes is reduced. *Klf4* expression is only reduced after RAD21^{AID} depletion, and expression of *Slco5a1* is specifically increased after CTCF^{AID} depletion, consistent with re-targeting of the enhancer to this gene after removal of CTCF binding in this locus[2].

b, Micro-C detected chromatin interactions in the indicated enhancer target loci in wild-type mESC, and weakening of chromatin loops after RAD21^{AID} depletion. Micro-C data are from reference[1].

Supplementary Fig. 3

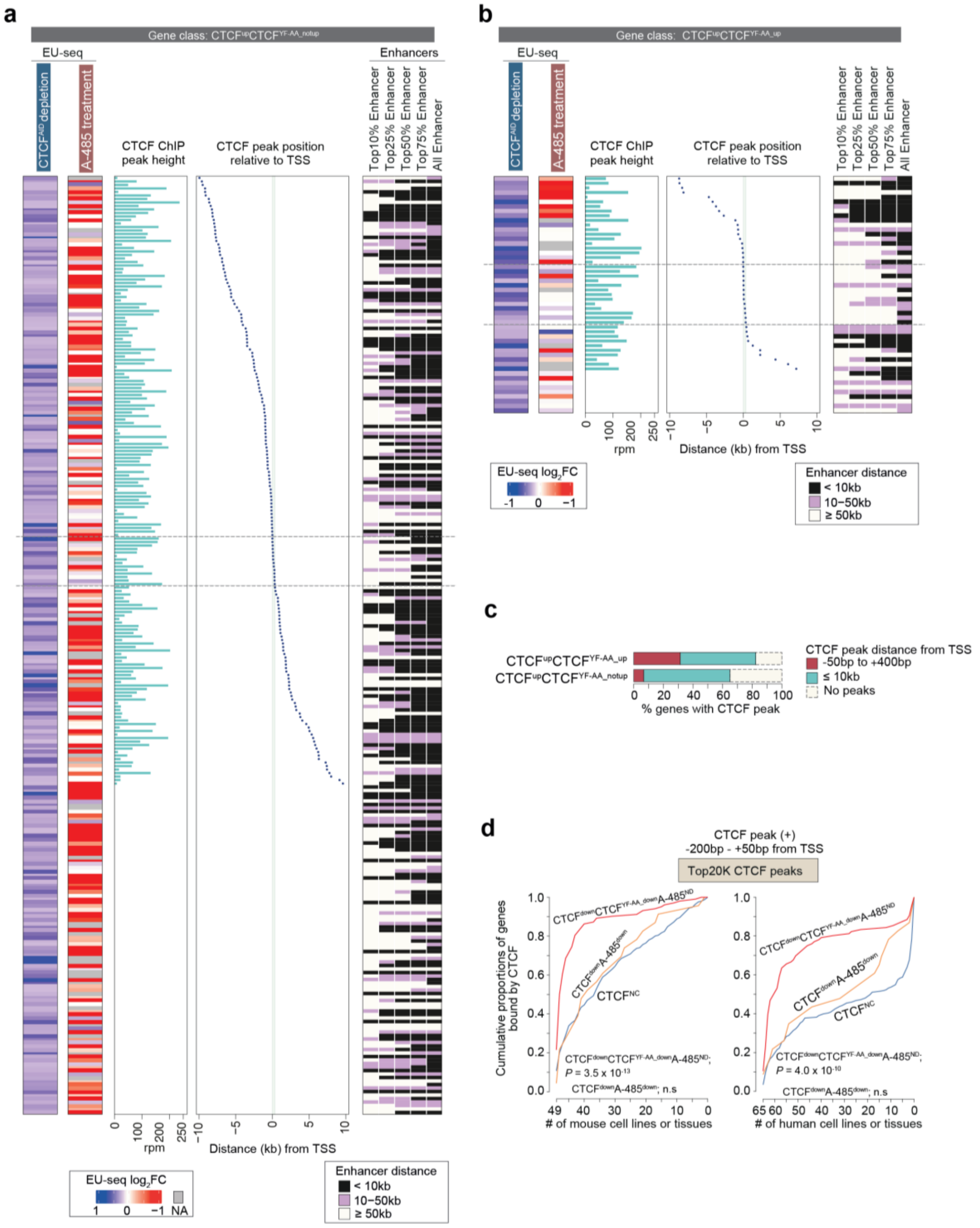

Supplementary Fig. 3. Promoter CTCF binding is broadly conserved in CTCF-regulated genes, and only a subset of genes upregulated by wild-type CTCF^{AID} depletion are upregulated by CTCF$^{YF\text{-}AA}$ depletion.

a, Properties of genes upregulated (>1.3 fold) after acute (6h) CTCF^{AID} depletion, but not upregulated after acute (2h) CTCF$^{YF\text{-}AA}$ depletion (CTCF^{up}CTCF$^{YF\text{-}AA_notup}$). For CTCF^{up}CTCF$^{YF\text{-}AA_notup}$ genes, the heatmap shows fold change in gene expression after CTCF^{AID} depletion and by A-485 treatment. CTCF peak position (+/-10kb from TSS) and peak height are indicated, along with the enrichment of candidate enhancers in their proximity. Regions from TSS to +400bp are highlighted. The genes between the dotted lines contain a CTCF peak located between TSS to +400bp. Candidate enhancers are defined as overlapping regions of

H2BK20ac peaks and H3K27ac peaks, excluding regions +/- 500bp from TSS. Enhancers are ranked by H2BK20ac ChIP signal enrichment.

b, Properties of genes commonly upregulated (>1.3-fold) after acute (6h) $CTCF^{AID}$ depletion and after acute (2h) $CTCF^{YF\text{-}AA}$ depletion ($CTCF^{up}CTCF^{YF\text{-}AA\_up}$). Shown is the fold increase in gene expression after $CTCF^{AID}$ depletion and regulation of the same genes by A-485 treatment. CTCF peak position (+/- 10kb from TSS) and peak height are indicated, along with the enrichment of candidate enhancers in their proximity. Regions from TSS to +400bp are highlighted. The genes between the dotted lines contain a CTCF peak located between TSS to +400bp. Candidate enhancers are defined as overlapping regions of H2BK20ac peaks and H3K27ac peaks, excluding regions +/- 500bp from TSS. Enhancers are ranked by H2BK20ac ChIP signal enrichment.

c, Fraction of genes showing CTCF binding within the indicated distance from TSS, for the indicated groups of regulated genes. Of note, $CTCF^{up}CTCF^{YF\text{-}AA\_up}$ genes exhibit a higher prevalence of CTCF binding in promoter overlapping and immediate downstream regions (-50bp to +400bp from TSS) than $CTCF^{up}CTCF^{YF\text{-}AA\_notup}$ genes.

d, Conservation of CTCF binding (-200bp to +50bp from TSS) in the promoters of the indicated classes of CTCF-regulated genes in mouse (left panel) and human (right panel) cell lines and tissues. Only genes bound by CTCF in promoters of mESC are included in this analysis. P-values were calculated using a two-sample Kolmogorov-Smirnov test.

Supplementary Fig. 4

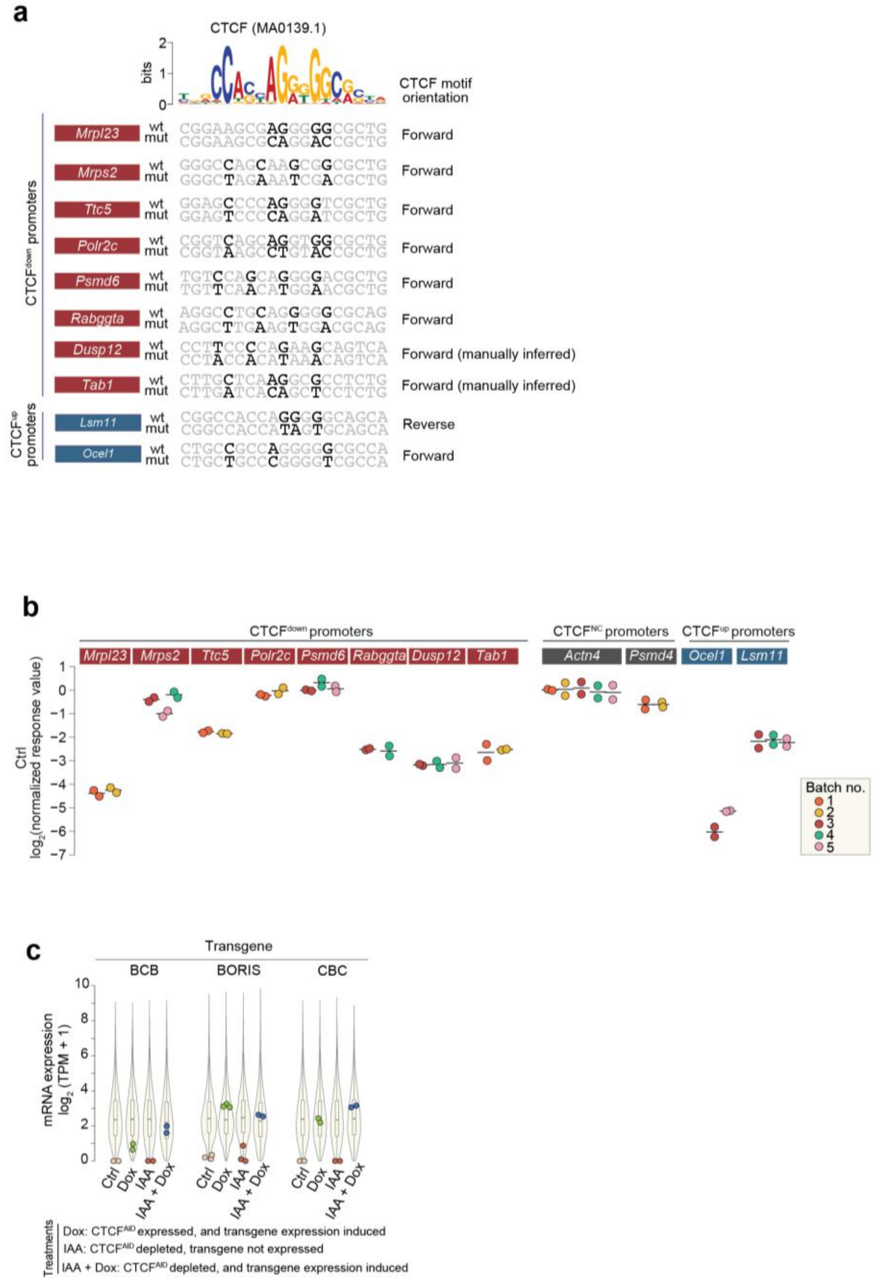

Supplementary Fig. 4. Functional and mechanistic consequence of promoter CTCF binding for gene regulation.

a, Displayed are sequences of putative CTCF binding motifs within the promoters of specified CTCF-regulated genes. The motif at the top represents the canonical CTCF binding motif. For *Dusp12* and *Tab1* promoters, computational analyses did not identify canonical motifs within CTCF peak regions; hence, the provided sequences represent manually inferred putative CTCF binding sequences. Nucleotides in dark black type denote mutations introduced to disrupt CTCF binding in the putative motifs. CTCF motif orientation is with respect to the orientation of the indicated genes in the genome.

b, Normalized luciferase activity for the indicated promoters. The basal promoter reporter activity is analyzed for wild-type promoter sequences in cells expressing $CTCF^{YF\text{-}AA}$ without the depletion of $CTCF^{YF\text{-}AA}$. Circles indicate measurements from individual experiments; the horizontal line indicates the median value. Biological replicates: n = 4 for *Mrpl23*, *Ttc5*, *Polr2c*, *Rabggta*, *Tab1*, *Psmd4*, *Lsm11*; n = 6 for *Mrps2*, *Psmd6*, *Dusp12*, *Ocel1*; n = 10 for *Actn4*.

c, Expression of the specified transgenes under designated treatment conditions. Doxycycline (Dox) treatment induces transgene expression, while indole-3-acetic acid (IAA) treatment depletes endogenous $CTCF^{AID}$ protein. All transgenes are *mRuby2*-fused. Transgene expression in the indicated treatment conditions is quantified by read counts mapping to *mRuby2* in RNA-seq data. The box plots display the median, upper and lower quartiles; the whiskers show the 1.5× interquartile range. CBC: CTCF-N-terminus_BORIS-ZF_CTCF-C-terminus, BCB: BORIS-N-terminus_CTCF-ZF_BORIS-C-terminus. Gene expression data sourced from reference[3].

Supplementary Tables

Supplementary Table 1

Genome sequences of homology donor sequences used for the generation of CRISPR-edited CTCF cell lines.

***Puro-P2A-Gfp-Fkbp^{12F36V}-Ctcf* homology donor sequence** (for generating ^{dTAG}CTCF cells)

XXX: Puro resistance marker, XXX: P2A, XXX: eGFP, XXX: FKBP12^{F36V}, XXX: homology arm

	Sequence
Homology donor sequence	GAAGCTAAGTTCTCTCAGTTGTAGCTTCAACTTTTCCTTGTTTTTTCTTTTGGCAGTTTATTCGCATAAGAAACCAAAATTAACATTGTAG<br>TGTTTCTCTTAGACTGAACTTCTGCAGTATATTTAACACCTGCTGTCACCAGTTTTCTGTTAATTTTGAGTATAATCTAGAGTCTTGATCA<br>GTTTCAGATTTTTTTGTTTTGATTGGCAAGATTTATAATATAGGTGGTGAATTTTTTTTTCTTCCATCAAAGAGGAAGATATCTATTAAAT<br>AACCATTGATGGATGCCTAATTCATTCACTAAGGTGGGTTTTTTGGGGGGTCAGGAGGTTGAGTTTTAGGGGTTTGGCTTGGTTTTTGGTT<br>CTTAGAGATAGGGTTTCTCTGTGCAGGCCAGGCTGGCCTCAAACTCAGAAATCTGCCTGCCTGTGGGTTCTAAGTGCTGGGATTAAAGGTG<br>TGTGCACCACTACCACCTGCCACTCAAATGATTTTAGTTTAAAATGTGTGCTTTGATTTGAGCAAACCACATCATAAATAACATCTGTGTT<br>CTCCTTAATAAAGGAAGGGGAGACCGGTACGCGTGCCACCatgaccgagtacaagcccacggtgcgcctcgccacccgcgacgacgtcccc<br>agggccgtacgcaccctcgccgccgcgttcgccgactaccccgccacgcgccacaccgtcgatccggaccgccacatcgagcgggtcaccg<br>agctgcaagaactcttcctcacgcgcgtcgggctcgacatcggcaaggtgtgggtcgcggacgacggcgccgcggtggcggtctggaccac<br>gccggagagcgtcgaagcgggggcggtgttcgccgagatcggcccgcgcatggccgagttgagcggttcccggctggccgcgcagcaacag<br>atggaaggcctcctggcgccgcaccggcccaaggagcccgcgtggttcctggccaccgtcggcgtctcgcccgaccaccagggcaagggtc<br>tgggcagcgccgtcgtgctccccggagtggaggcggccgagcgcgccggggtgcccgccttcctggagacctccgcgccccgcaacctccc<br>cttctacgagcggctcggcttcaccgtcaccgccgacgtcgaggtgcccgaaggaccgcgcacctggtgcatgacccgcaagcccggtgcc<br>GGAAGCGGAGCTACTAACTTCAGCCTGCTGAAGCAGGCTGGAGACGTGGAGGAGAACCCTGGACCTCTTAAGATGGTGAGCAAGGGCGAGG<br>AGCTGTTCACCGGGGTGGTGCCCATCCTGGTCGAGCTGGACGGCGACGTAAACGGCCACAAGTTCAGCGTGTCCGGCGAGGGCGAGGGCGA<br>TGCCACCTACGGCAAGCTGACCCTGAAGTTCATCTGCACCACCGGCAAGCTGCCCGTGCCCTGGCCCACCCTCGTGACCACCCTGACCTAC<br>GGCGTGCAGTGCTTCAGCCGCTACCCCGACCACATGAAGCAGCACGACTTCTTCAAGTCCGCCATGCCCGAAGGCTACGTCCAGGAGCGCA<br>CCATCTTCTTCAAGGACGACGGCAACTACAAGACCCGCGCCGAGGTGAAGTTCGAGGGCGACACCCTGGTGAACCGCATCGAGCTGAAGGG<br>CATCGACTTCAAGGAGGACGGCAACATCCTGGGGCACAAGCTGGAGTACAACTACAACAGCCACAACGTCTATATCATGGCCGACAAGCAG<br>AAGAACGGCATCAAGGTGAACTTCAAGATCCGCCACAACATCGAGGACGGCAGCGTGCAGCTCGCCGACCACTACCAGCAGAACACCCCCA<br>TCGGCGACGGCCCCGTGCTGCTGCCCGACAACCACTACCTGAGCACCCAGTCCGCCCTGAGCAAAGACCCCAACGAGAAGCGCGATCACAT<br>GGTCCTGCTGGAGTTCGTGACCGCCGCCGGGATCACTCTCGGCATGGACGAGCTGTACAAGGGTGGAGGCGGTAGTGGTGGAGGCGGTAGT<br>GGTATGGGAGTGCAGGTGGAAACCATCTCCCCAGGAGACGGGCGCACCTTCCCCAAGCGCGGCCAGACCTGCGTGGTGCACTACACCGGGA<br>TGCTTGAAGATGGAAAGAAAGTTGATTCCTCCCGGGACAGAAACAAGCCCTTTAAGTTTATGCTAGGCAAGCAGGAGGTGATCCGAGGCTG<br>GGAAGAAGGGGTTGCCCAGATGAGTGTGGGTCAGAGAGCCAAACTGACTATATCTCCAGATTATGCCTATGGTGCCACTGGGCACCCAGGC<br>ATCATCCCACCACATGCCACTCTCGTCTTCGATGTGGAGCTTCTAAAACTGGAAGCTAGCCATATGCTGCAGATGGAAGGTGAGGCGGTTG<br>AAGCCATTGTGGAGGAGTCTGAAACTTTCATTAAAGGAAAAGAAAGAAAGACTTACCAGAGACGCCGGGAAGGGGGCCAGGAAGAAGATGC<br>TTGCCACCTGCCCCAGAACCAGACAGATGGGGGTGAGGTGGTCCAGGATGTCAACAGCAGTGTACAGATGGTAATGATGGAACAGCTGGAT<br>CCTACCCTTCTCCAGATGAAGACTGAAGTCATGGAGGGTACAGTGGCTCCGGAAGCAGAGGCTGCAGTGGACGATACCCAGATCATAACCT<br>TGCAGGTTGTAAATATGGAGGAACAGCCCATTAACATAGGAGAGCTTCAGCTTGTCCAAGTACCTGTTCCTGTGACGGTACCTGTTGCTAC<br>TACTTCAGTAGAAGAACTTCAGGGGGCTTATGAGAATGAAGTGTCTAAAGAGGGCCTTGCAGAAAGTGAACCGATGATATGTCACACCTTA<br>CCTTTGCCTGAAGGATTTCAGGTGGTGAAAGTGGGGGCCAATGGAGAAGTGGAGACACTAGAGCAGGGCGAGCTTCCTCCTCAGGAAGACT<br>CTAGCTGGCAAAAAGACCCAGACTATCAGCCACCAGCCAAAAAAACAAAGAAAACCAAAAAGAGCAAACTTCGTTACACAGAAGAGGGCAA<br>AGACGTGGATGTGTCTGTGTATGATTTTGAGGAAGAACAGCAGGAAGGACTGCTGTCTGAGGTTAATGCAGAGAAAGTAGTTGGTAATATG<br>AAGCCTCCGAAGCCAACAAAAATTAAAAAAAAAGGTAAAGTGAGTTTATCTCTAATGGACTTGTACAACCATTTGGGAAAAGGATACAAC<br>AGCCTCTATGCAAGTTATTTTGTCAAAAAAAAAATTTTTTTAAATGTCTAGTGGGTGCCATGGCTTTTAATCAGTCCAAAAGAAGGGTTTT<br>ATTTATGTTTTTAAGTTCTCAAGAGAAGTGAGTATTAATTCTGTGAATTAATTTCTACTTAGATTGACTTTGATTATAAAAAGTTAAGAGA<br>ATATCATTGTAGTTTGGGATTAATCTAAACATTGAAGCACTGTAACAAGTCTTATGTTTTGTTTTGCATATAGGTGTAAAGAAAACATTCC<br>AGTGTGAGCTTTGCAGTTACACATGTCCCCGGCGTTCAAATTTGGATCGTCACATGAAAAGCCACACTGATGAGAGACCACACAAATGCCA<br>TCTGTGTGGCAGAGCATTCAGAACAGTGACCCTCCTGAGGAATCATCTGAACACACACACAGGTGCTGCATAGTAATGT
CRISPR gRNA oligos for PX330	Guide oligo1: CACCGGAAGGGGAGATGGAAGGTG
	Guide oligo2: CACCAGGGGAGATGGAAGGTGAGG

***Puro-P2A-Gfp-Fkbp^{12F36V}-Ctcf$^{Y22A6/F228A}$* homology donor sequence** (for generating CTCF^{YF-AA} cells)

XXX: Puro resistance marker, XXX: P2A, XXX: eGFP, XXX: FKBP12^{F36V}, XXX: homology arm (mutated nucleotides are underlined)

	Sequence
Homology donor sequence	GAAGCTAAGTTCTCTCAGTTGTAGCTTCAACTTTTCCTTGTTTTTTCTTTTGGCAGTTTATTCGCATAAGAAACCAAAATTAACATTGTAG<br>TGTTTCTCTTAGACTGAACTTCTGCAGTATATTTAACACCTGCTGTCACCAGTTTTCTGTTAATTTTGAGTATAATCTAGAGTCTTGATCA<br>GTTTCAGATTTTTTTGTTTTGATTGGCAAGATTTATAATATAGGTGGTGAATTTTTTTTTCTTCCATCAAAGAGGAAGATATCTATTAAAT<br>AACCATTGATGGATGCCTAATTCATTCACTAAGGTGGGTTTTTTGGGGGGTCAGGAGGTTGAGTTTTAGGGGTTTGGCTTGGTTTTTGGTT<br>CTTAGAGATAGGGTTTCTCTGTGCAGGCCAGGCTGGCCTCAAACTCAGAAATCTGCCTGCCTGTGGGTTCTAAGTGCTGGGATTAAAGGTG<br>TGTGCACCACTACCACCTGCCACTCAAATGATTTTAGTTTAAAATGTGTGCTTTGATTTGAGCAAACCACATCATAAATAACATCTGTGTT<br>CTCCTTAATAAAGGAAGGGGAGACCGGTACGCGTGCCACCatgaccgagtacaagcccacggtgcgcctcgccacccgcgacgacgtcccc<br>agggccgtacgcaccctcgccgccgcgttcgccgactaccccgccacgcgccacaccgtcgatccggaccgccacatcgagcgggtcaccg<br>agctgcaagaactcttcctcacgcgcgtcgggctcgacatcggcaaggtgtgggtcgcggacgacggcgccgcggtggcggtctggaccac<br>gccggagagcgtcgaagcgggggcggtgttcgccgagatcggcccgcgcatggccgagttgagcggttcccggctggccgcgcagcaacag<br>atggaaggcctcctggcgccgcaccggcccaaggagcccgcgtggttcctggccaccgtcggcgtctcgcccgaccaccagggcaagggtc<br>tgggcagcgccgtcgtgctccccggagtggaggcggccgagcgcgccggggtgcccgccttcctggagacctccgcgccccgcaacctccc<br>cttctacgagcggctcggcttcaccgtcaccgccgacgtcgaggtgcccgaaggaccgcgcacctggtgcatgacccgcaagcccggtgcc<br>GGAAGCGGAGCTACTAACTTCAGCCTGCTGAAGCAGGCTGGAGACGTGGAGGAGAACCCTGGACCTCTTAAGATGGTGAGCAAGGGCGAGG<br>AGCTGTTCACCGGGGTGGTGCCCATCCTGGTCGAGCTGGACGGCGACGTAAACGGCCACAAGTTCAGCGTGTCCGGCGAGGGCGAGGGCGA<br>TGCCACCTACGGCAAGCTGACCCTGAAGTTCATCTGCACCACCGGCAAGCTGCCCGTGCCCTGGCCCACCCTCGTGACCACCCTGACCTAC<br>GGCGTGCAGTGCTTCAGCCGCTACCCCGACCACATGAAGCAGCACGACTTCTTCAAGTCCGCCATGCCCGAAGGCTACGTCCAGGAGCGCA<br>CCATCTTCTTCAAGGACGACGGCAACTACAAGACCCGCGCCGAGGTGAAGTTCGAGGGCGACACCCTGGTGAACCGCATCGAGCTGAAGGG<br>CATCGACTTCAAGGAGGACGGCAACATCCTGGGGCACAAGCTGGAGTACAACTACAACAGCCACAACGTCTATATCATGGCCGACAAGCAG<br>AAGAACGGCATCAAGGTGAACTTCAAGATCCGCCACAACATCGAGGACGGCAGCGTGCAGCTCGCCGACCACTACCAGCAGAACACCCCCA<br>TCGGCGACGGCCCCGTGCTGCTGCCCGACAACCACTACCTGAGCACCCAGTCCGCCCTGAGCAAAGACCCCAACGAGAAGCGCGATCACAT

	GGTCCTGCTGGAGTTCGTGACCGCCGCCGGGATCACTCTCGGCATGGACGAGCTGTACAAGGGTGGAGGCGGTAGTGGTGGAGGCGGTAGT<br>GGTATGGGAGTGCAGGTGGAAACCATCTCCCCAGGAGACGGGCGCACCTTCCCCAAGCGCGGCCAGACCTGCGTGGTGCACTACACCGGGA<br>TGCTTGAAGATGGAAAGAAAGTTGATTCCTCCCGGGACAGAAACAAGCCCTTTAAGTTTATGCTAGGCAAGCAGGAGGTGATCCGAGGCTG<br>GGAAGAAGGGGTTGCCCAGATGAGTGTGGGTCAGAGAGCCAAACTGACTATATCTCCAGATTATGCCTATGGTGCCACTGGGCACCCAGGC<br>ATCATCCCACCACATGCCACTCTCGTCTTCGATGTGGAGCTTCTAAAACTGGAAGCTAGCCATATGCTGCAGATGGAAGGTGAGGCGGTTG<br>AAGCCATTGTGGAGGAGTCTGAAACTTTCATTAAAGGAAAAGAAAGAAAGACTTACCAGAGACGCCGGGAAGGGGGCCAGGAAGAAGATGC<br>TTGCCACCTGCCCCAGAACCAGACAGATGGGGGTGAGGTGGTCCAGGATGTCAACAGCAGTGTACAGATGGTAATGATGGAACAGCTGGAT<br>CCTACCCTTCTCCAGATGAAGACTGAAGTCATGGAGGGTACAGTGGCTCCGGAAGCAGAGGCTGCAGTGGACGATACCCAGATCATAACCT<br>TGCAGGTTGTAAATATGGAGGAACAGCCCATTAACATAGGAGAGCTTCAGCTTGTCCAAGTACCTGTTCCTGTGACGGTACCTGTTGCTAC<br>TACTTCAGTAGAAGAACTTCAGGGGGCTTATGAGAATGAAGTGTCTAAAGAGGGCCTTGCAGAAAGTGAACCGATGATATGTCACACCTTA<br>CCTTTGCCTGAAGGATTTCAGGTGGTGAAAGTGGGGGCCAATGGAGAAGTGGAGACACTAGAGCAGGGCGAGCTTCCTCCTCAGGAAGACT<br>CTAGCTGGCAAAAAGACCCAGACTATCAGCCACCAGCCAAAAAAACAAAGAAAACCAAAAAGAGCAAACTTCGTTACACAGAAGAGGGCAA<br>AGACGTGGATGTGTCTGTG**gcc**GAT**gct**GAGGAAGAACAGCAGGAAGGACTGCTGTCTGAGGTTAATGCAGAGAAAGTAGTTGGTAATATG<br>AAGCCTCCGAAGCCAACAAAAATTAAAAAAAAAGGTAAAGTGAGTTTATCTCTAATGGACTTGTACAACCATTTGGGAAAAGGATACAAC<br>AGCCTCTATGCAAGTTATTTTGTCAAAAAAAAAATTTTTTTAAATGTCTAGTGGGTGCCATGGCTTTTAATCAGTCCAAAAGAAGGGTTTT<br>ATTTATGTTTTTAAGTTCTCAAGAGAAGTGAGTATTAATTCTGTGAATTAATTTCTACTTAGATTGACTTTGATTATAAAAAGTTAAGAGA<br>ATATCATTGTAGTTTGGGATTAATCTAAACATTGAAGCACTGTAACAAGTCTTATGTTTTGTTTTGCATATAGGTGTAAAGAAAACATTCC<br>AGTGTGAGCTTTGCAGTTACACATGTCCCCGGCGTTCAAATTTGGATCGTCACATGAAAAGCCACACTGATGAGAGACCACACAAATGCCA<br>TCTGTGTGGCAGAGCATTCAGAACAGTGACCCTCCTGAGGAATCATCTGAACACACACACAGGTGCTGCATAGTAATGT
CRISPR gRNA oligos for PX330	Guide oligo1: CACCGTGTGTCTGTGTATGATTTTG
	Guide oligo2: CACCCTGATTTTGAGGAAGAACAGC
	Guide oligo3: CACCGGAAGGGGAGATGGAAGGTG
	Guide oligo4: CACCAGGGGAGATGGAAGGTGAGG

***Hygro-P2A-3XFLAG-BromoTag-*ΔN*Ctcf* homology donor sequence** (for generating $^{\text{bTAG_}\Delta\text{N}}$CTCF)

XXX: Hygomycin resistance marker, XXX: P2A, XXX: 3XFLAG, XXX: BromoTag XXX: homology arm

	Sequence
Homology donor sequence	CTGCGGCCGCACGGATCCTCGAATTagaagctaagttctctcagttgtagcttcaacttttccttgtttttcttttggcagtttattcgc<br>ataagaaaccaaaattaacattgtagtgtttctcttagactgaacttctgcagtatatttaacacctgctgtcaccagttttctgttaatt<br>ttgagtataatctagagtcttgatcagtttcagatttttttgttttgattggcaagatttataatataggtggtgaattttttttttcttcc<br>atcaaagaggaagatatctattaaataaccattgatggatgcctaattcattcactaaggtgggtttttggggggtcaggaggttgagtt<br>ttaggggtttggcttggtttttggttcttagagatagggtttctctgtgcaggccaggctggcctcaaactcagaaatctgcctgcctgtg<br>ggttctaagtgctgggattaaaggtgtgtgcaccactaccacctgccactcaaatgattttagtttaaaatgtgtgctttgatttgagcaa<br>accacatcataaataacatctgtgttctccttaataaagGAAGGGGAGACCGGTACGCGTGCCACCATGAAAAAGCCTGAACTCACCGCGA<br>CGTCTGTCGAGAAGTTTCTGATCGAAAAGTTCGACAGCGTCTCCGACCTGATGCAGCTCTCGGAGGGCGAAGAATCTCGTGCTTTCAGCTT<br>CGATGTAGGAGGGCGTGGATATGTCCTGCGGGTAAATAGCTGCGCCGATGGTTTCTACAAAGATCGTTATGTTTATCGGCACTTTGCATCG<br>GCCGCGCTCCCGATTCCGGAAGTGCTTGACATTGGGGAATTCAGCGAGAGCCTGCCTATTGCATCTCCCGCCGTGCACAGGGTGTCACGTT<br>GCAAGACCTGCCTGAAACCGAACTGCCCGCTGTTCTACAGCCGGTCGCGGAGGCCATGGATGCGATCGCTGCGGCCGATCTTAGCCAGACG<br>AGCGGGTTCGGCCCATTCGGACCGCAAGGAATCGGTCAATACACTACATGGCGTGATTTCATCTGCGCGATTGCTGATCCCCATGTGTATC<br>ACTGGCAAACTGTGATGGACGACACCGTCAGTGCGTCCGTCGCGCAGGCTCTCGATGAGCTGATGCTTTGGGCCGAGGACTGCCCCGAAGT<br>CCGGCACCTCGTGCACGCGGATTTCGGCTCCAACAATGTCCTGACGGACAATGGCCGCATAACAGCGGTCATTGACTGGAGCGAGGCGATG<br>TTCGGGGATTCCCAATACGAGGTCGCCAACATCTTCTTCTGGAGGCCGTGGTTGGCTTGTATGGAGCAGCAGACGCGCTACTTCGAGCGGA<br>GGCATCCGGAGCTTGCAGGATCGCCGCGGCTCCGGGCGTATATGCTCCGCATTGGTCTTGACCAACTCTATCAGAGCTTGGTTGACGGCAA<br>TTTCGATGATGCAGCTTGGGCGCAGGGTCGATGCGACGCAATCGTCCGATCCGGAGCCGGGACTGTCGGGCGTACACAAATCGCCCGCAGA<br>AGCGCGGCCGTCTGGACCGATGGCTGTGTAGAAGTACTCGCCGATAGTGGAAACCGACGCCCCAGCACTCGTCCGAGGGCAAAGGAAGGAA<br>GCGGAGCTACTAACTTCAGCCTGCTGAAGCAGGCTGGAGACGTGGAGGAGAACCCTGGACCTCTTAAGATGGACTACAAGGATGATGATGA<br>CAAGGATTACAAGGACGACGACGACAAGGATTACAAGGATGATGATGACAAGGGCAGCGTGAAGGACGTGCCCGACTCTCAGCAGCACCCA<br>GCACCAGAGAAGAGCAGCAAGGTCTCGGAGCAGCTCAAGTGCTGCAGCGGCATCCTCAAGGAGATGTTTGCCAAGAAGCACGCCGCCTACG<br>CCTGGCCCTTCTACAAGCCTGTGGACGTGGAGGCACTGGGCGCCCACGACTACTGTGACATCATCAAGCACCCCATGGACATGAGCACAAT<br>CAAGTCTAAACTGGAGGCCCGTGAGTACCGTGATGCTCAGGAGTTTGGTGCTGACGTCCGATTGATGTTCTCCAACTGCTATAAGTACAAC<br>CCTCCTGACCATGAGGTGGTGGCCATGGCCCGCAAGCTCCAGGATGTGTTCGAAATGCGCTTTGCCAAGATGCCGGACGAGGGcGGtGGaG<br>GaAGcGGaGGcGGtGGcAGTGGaGGtGGCGGTAGTGCTAGCCATATGCTGCAGTTCCAGTGTGAGCTTTGCAGTTACACATGTCCCCGGCG<br>TTCAAATTTGGATCGTCACATGAAAAGCCACACTGATGAGAGACCACACAAATGCCATCTGTGTGGCAGAGCATTCAGAACAGTGACCCTC<br>CTGAGGAATCATCTGAACACACACACAGgtgctgcatagtaatgtgggctacggcatggtaaaggttaaaactcggttcctaattcacgat<br>tactgcagtaagaagttgaagaatattatttttgtttcttcttgtaacctgcagtttttctctgacataattcataaagattttataacat<br>tatgttgttaaggattggtagaatttgtcagtgtgtacacattgacatgagtactctgtcttaaactgtgcttaagctgtgaaaagttcat<br>tctgtggttgtccagacttggtaaaacttactcacctagttactcacctagttaagccagcaaaagtcaaatgcgcacatattgagtgcca<br>cttccgatgaagataagcttttcatcttctaaatgtgcttatattacattctaggaggcattctttggattgtttgttttactaagacaga<br>gtcttctacatagccctgTTGGATCCCAGAA
CRISPR gRNA oligos for PX330	Guide oligo1: CGTGGGCTTGTACTCGGTCA
	Guide oligo2: CATGACCGAGTACAAGCCCA

***Ctcf-ALFA-tag-P2A-Neo-Stop codon-polyA* homology donor sequence** (for modifying ^{dTAG}CTCF to generate ^{dTAG}CTCF$^{\Delta\text{C}}$)

XXX: Neomycin resistance marker, XXX: P2A, XXX: ALFA-tag, XXX: polyA tail, XXX: homology arm

	Sequence
Homology donor sequence	GGATCCTCGAATtctttatgagagttgaaactgtcaaatatttagaaataagatgacttgagtaggctgagagtaaaaaggcatttccttg<br>cgtggattgaaatctgtatttcatcctatccctaattaatctgactcggctctctctgttgattagccaagtcaacactaaccagctattc<br>tgaaactcccccaagtttctgtgtgactacaggcactgagctcaaacccttccccagccccaaccccagccccccatacacccatgtgcac<br>ggggcccagccttcttactggtgcttcctcgctcctttcctctttctgtttcctcccccatccctgatgactacagatgctgtgcatcttt<br>cctgaatggatgggaggaacatgttgagtacagttagcagaattttgagactttatggattccttaaagtttgtttttgttcagttaaaga<br>gaactttgagtctgaactttgttcttttagtagtgtgaaacaaggtttcttccttttgtcagAACACAATGGCAAGACATGCAGATAACTG<br>TACGCGTGCCACCAGCAGGTTAGAGGAGGAGTTAAGGCGTAGGCTCACCGAGGCTACTAACTTCAGCCTGCTGAAGCAGGCTGGAGACGTG<br>GAGGAGAACCCTGGACCTATGATTGAACAAGATGGCCTACATGCAGGTTCTCCAGCTGCCTGGGTTGAGAGACTGTTTGGCTATGACTGGG<br>CACAGCAGACCATTGGTTGCTCTGATGCAGCAGTTTTCAGACTTTCAGCCCAAGGCAGGCCAGTCCTTTTTGTAAAGACAGACCTCAGTGG<br>GGCTCTCAATGAGCTCCAGGATGAGGCTGCCAGACTCTCCTGGTTGGCAACAACTGGGGTCCCCTGTGCAGCTGTCCTTGATGTGGTCACA<br>GAAGCTGGAAGGGACTGGCTCCTACTAGGTGAGGTGCCTGGGCAGGACCTCCTTTCCTCTCACCTAGCTCCAGCTGAGAAAGTGTCAATCA<br>TGGCTGATGCCATGAGAAGACTCCACACCCTTGACCCAGCCACCTGCCCCTTTGACCACCAGGCCAAGCACAGGATAGAGAGGGCCAGAAC<br>CAGGATGGAGGCTGGCCTGGTGGACCAAGATGACTTGGATGAAGAACACCAGGGCCTGGCCCCTGCTGAACTATTTGCCAGGCTCAAGGCA

	TCCATGCCAGATGGTGAGGACCTAGTGGTGACTCATGGGGATGCCTGCCTTCCCAACATCATGGTTGAAAATGGAAGGTTCTCTGGCTTCA<br>TAGACTGTGGCAGGCTGGGAGTGGCTGACAGGTACCAGGACATTGCCCTAGCAACCAGGGACATAGCAGAAGAGCTAGGGGGAGAGTGGGC<br>AGACAGGTTCCTAGTGCTCTATGGCATTGCAGCCCCTGACTCCCAGAGAATTGCCTTCTACAGACTTCTTGATGAGTTCTTCTAATCTAGA<br>GGGCCCGTTTAAACCCGCTGATCAGCCTCGActgtgccttctagttgccagccatctgttgtttgcccctcccccgtgccttccttgaccc<br>tggaaggtgccactcccactgtcctttcctaataaaatgaggaaattgcatcgcattgtctgagtaggtgtcattctattctggggggtgg<br>ggtggggcaggacagcaagggggaggattgggaagacaatagcaggcatgctggggatgcggtgggctctatgggtcgacGCTAGCCATAT<br>GCTGCAGTGACTGATTAAGCTGGTCCAGATGGCGTAGAGGGGGAAAATGGAGGGGAGACAAAGAAGAGCAAACGAGGAAGAAAAAGAAAGA<br>TGCGATCTAAAAAAGAAGACTCCTCTGACAGTGgtaagtgggtcattgttgatttggttagaggtgacacatcagagacccagttttcagt<br>aaatctgtgtaaggtagtagaggcaagaaggcccctctcctttatgcttggaatctttctaaagccaggtgctactcacggaggggggaagc<br>cggaagagcaaagcagagttcccatgccagactctccttagcaagtgcaaggctagccttggcatctgaaatgtcagatgagcccccgaag<br>ttagaagtgatgacataagcatgaggttgcagagctgtgtagtttgctgcagtgctccctgctgctgtagactctgcattgtcttctcttt<br>ccctgctacctaccatgcccttcattatgttcatgttctctctctgaagaaactactgacagcctctactagatctgtactgttctttctt<br>ctgacttcaccgctggtttcaTTGGATCCCAGAA
CRISPR gRNA oligo for PX330	Guide oligo1: AGACATGCAGATAACTGTGC

Supplementary Table 2

Promoter sequences used in the CTCF promoter luciferase assay.

XXXX: CTCF motif, XXXX: mutated sequence,

Gene	CTCF motif	Sequence	Length
Polr2c	wt	AAATTATTTTTATGTATAAGGATGTTTTGCTTGCATGTATATTTGTGCACCATGTGAATACCTGGTGCCCTC<br>AGATACCAGGAGAGAGTTTCCTCAGAAACTGAAGCTAAAGATGTTAGGCATCATATGGGTGCTGGGAATACA<br>ATCCAAGTCTTACATAAGAGCAGCCAGTGCTCTTATCCCCAATGCCGTATCTCCAGCCCCGTATGTAAGTTT<br>AACCAAGTCTCTATCGATCATTGCCTAATGCGTTATTTTTAGCAGTTATAAATAACGTTGGCATGAATGACC<br>CTGTACATTTAATTTTTGTGCTGTATATCTTGGGGATTTCAAGAGTATAGGACCAATGCATTTTGGCGCCGG<br>TGTGGTTAGAAAACACAGGCCAGGAGAGTTCCGGCCTCCGGTCAGCAGGTGGCGCTGCGCCCGCGACTAGCC<br>GGAAGCGCCGATTGCCCCGCCGACAAACTGCTGGTGGCCGCGCAGGCGCGCTGTGCGGCTGGCGCGGGAGCC<br>TGGCGGAGGGTCGCGATGCCGTACGCCAACCAGCCAACAGTGCGGA	550bp
	mut	AAATTATTTTTATGTATAAGGATGTTTTGCTTGCATGTATATTTGTGCACCATGTGAATACCTGGTGCCCTC<br>AGATACCAGGAGAGAGTTTCCTCAGAAACTGAAGCTAAAGATGTTAGGCATCATATGGGTGCTGGGAATACA<br>ATCCAAGTCTTACATAAGAGCAGCCAGTGCTCTTATCCCCAATGCCGTATCTCCAGCCCCGTATGTAAGTTT<br>AACCAAGTCTCTATCGATCATTGCCTAATGCGTTATTTTTAGCAGTTATAAATAACGTTGGCATGAATGACC<br>CTGTACATTTAATTTTTGTGCTGTATATCTTGGGGATTTCAAGAGTATAGGACCAATGCATTTTGGCGCCGG<br>TGTGGTTAGAAAACACAGGCCAGGAGAGTTCCGGCCTCCGGTAAGCCTGTACCGCTGCGCCCGCGACTAGCC<br>GGAAGCGCCGATTGCCCCGCCGACAAACTGCTGGTGGCCGCGCAGGCGCGCTGTGCGGCTGGCGCGGGAGCC<br>TGGCGGAGGGTCGCGATGCCGTACGCCAACCAGCCAACAGTGCGGA	550bp
	inv	AAATTATTTTTATGTATAAGGATGTTTTGCTTGCATGTATATTTGTGCACCATGTGAATACCTGGTGCCCTC<br>AGATACCAGGAGAGAGTTTCCTCAGAAACTGAAGCTAAAGATGTTAGGCATCATATGGGTGCTGGGAATACA<br>ATCCAAGTCTTACATAAGAGCAGCCAGTGCTCTTATCCCCAATGCCGTATCTCCAGCCCCGTATGTAAGTTT<br>AACCAAGTCTCTATCGATCATTGCCTAATGCGTTATTTTTAGCAGTTATAAATAACGTTGGCATGAATGACC<br>CTGTACATTTAATTTTTGTGCTGTATATCTTGGGGATTTCAAGAGTATAGGACCAATGCATTTTGGCGCCGG<br>TGTGGTTAGAAAACACAGGCCAGGAGAGTTCCGGCCTCCAGCGCCACCTGCTGACCGCGCCCGCGACTAGCC<br>GGAAGCGCCGATTGCCCCGCCGACAAACTGCTGGTGGCCGCGCAGGCGCGCTGTGCGGCTGGCGCGGGAGCC<br>TGGCGGAGGGTCGCGATGCCGTACGCCAACCAGCCAACAGTGCGGA	550bp
Psmd6	wt	CATGTGCACACAAAAATAATGTAAATCACTGAATTAATAATTGGAAACACGGGGTACTCTTTTCTTAGAATA<br>GGGAAGAATGTGGATTTTAAATGTAAGGAAGATAGGTTCTACACAGGCCTTGTCATGTAGCTGGTGTGTAAC<br>CTTAGCTGGCCACTTTTCAAATCTCGCTATGTTCCAGTTTGTTTGTCTACAATCTGGACCAAAAGAATCACT<br>CCCTAGTTCAAGAGTATTGAAACAAAGATGTCCCTGGAAAACTGTTTTTATTGTTTTTCTCATCTGTGTTAT<br>ATTTTAAAACTGTTGGCGGATTTCCAGATCCTATCCGTTGCAAATATTATATAAAAAGGAAGAAAAAAAGAT<br>ACTCAAGAAATGAAAGTTGGGCCCAACTCTGACCTAAAGAAAAAGGGGACAGCGCCAGGTGCTCCCGCCGAT<br>GTCCAGCAGGGGACGCTGCGGGCGGCGGAGCCAGGGACGCGACCGGAAGTCAGACTGGCGGCTCTGGGGAAC<br>CTGTGACGACTCCTCCTGCTTCCGCTGGTGGCCCAGGGCTGTGCTT	550bp
	mut	CATGTGCACACAAAAATAATGTAAATCACTGAATTAATAATTGGAAACACGGGGTACTCTTTTCTTAGAATA<br>GGGAAGAATGTGGATTTTAAATGTAAGGAAGATAGGTTCTACACAGGCCTTGTCATGTAGCTGGTGTGTAAC<br>CTTAGCTGGCCACTTTTCAAATCTCGCTATGTTCCAGTTTGTTTGTCTACAATCTGGACCAAAAGAATCACT<br>CCCTAGTTCAAGAGTATTGAAACAAAGATGTCCCTGGAAAACTGTTTTTATTGTTTTTCTCATCTGTGTTAT<br>ATTTTAAAACTGTTGGCGGATTTCCAGATCCTATCCGTTGCAAATATTATATAAAAAGGAAGAAAAAAAGAT<br>ACTCAAGAAATGAAAGTTGGGCCCAACTCTGACCTAAAGAAAAAGGGGACAGCGCCAGGTGCTCCCGCCGAT<br>GTTCAACATGGAACGCTGCGGGCGGCGGAGCCAGGGACGCGACCGGAAGTCAGACTGGCGGCTCTGGGGAAC<br>CTGTGACGACTCCTCCTGCTTCCGCTGGTGGCCCAGGGCTGTGCTT	550bp
	inv	CATGTGCACACAAAAATAATGTAAATCACTGAATTAATAATTGGAAACACGGGGTACTCTTTTCTTAGAATA<br>GGGAAGAATGTGGATTTTAAATGTAAGGAAGATAGGTTCTACACAGGCCTTGTCATGTAGCTGGTGTGTAAC<br>CTTAGCTGGCCACTTTTCAAATCTCGCTATGTTCCAGTTTGTTTGTCTACAATCTGGACCAAAAGAATCACT<br>CCCTAGTTCAAGAGTATTGAAACAAAGATGTCCCTGGAAAACTGTTTTTATTGTTTTTCTCATCTGTGTTAT<br>ATTTTAAAACTGTTGGCGGATTTCCAGATCCTATCCGTTGCAAATATTATATAAAAAGGAAGAAAAAAAGAT<br>ACTCAAGAAATGAAAGTTGGGCCCAACTCTGACCTAAAGAAAAAGGGGACAGCGCCAGGTGCTCCCGCCGAC<br>AGCGTCCCCTGCTGGACACGGGCGGCGGAGCCAGGGACGCGACCGGAAGTCAGACTGGCGGCTCTGGGGAAC<br>CTGTGACGACTCCTCCTGCTTCCGCTGGTGGCCCAGGGCTGTGCTT	550bp
Rabggta	wt	CTAAAAACGTTAGATTGAGTTCCTAAAGCTAGCTACCGAGATCCGTTCCCTTATTCACCTCATCCTAACACA<br>GGATTTCCCCTTTTACCTTTATAAACTGCCACTCTCCTATGGGCCATGCCTGTCTCCTCTCTATTCAAAGAC<br>AGTCCTTTGTCCCCTCTTCGAGAGAAATACCTGCCCCCCCGTCCCCCCCCTCCCCTCTTCTCTTTCCCCTT<br>CTCCATCTCCTGTCTTGTCTCTTATTCCTATACTTCGTCCCTCTAGGGCAAATAAAGCTCCTTTGTCCTGAA<br>AACTTGGTCTTGGGGTGTCCTGAGCCGATACCAGTCCCTTCAAGGCTGCAGTTTGAGTTGCGAATGGGAAGA<br>GCCAGGCCTGCAGGGGGCGCAGCGCCTCGTCTCCGCACCTCACACAGCACGGAGCCGCTCTAGCCACACTGG<br>GACTCGGTGGCGGCCGGAGTTGCACGTCCTGGCACCTCCTCCCTGAGCAGGTGAGTTCGCCGGTGGAGGAGG<br>GGGTGCAAGGGTCCACGGGACGGACGGACGGGCGGGCGGGCACTGCT	551bp
	mut	CTAAAAACGTTAGATTGAGTTCCTAAAGCTAGCTACCGAGATCCGTTCCCTTATTCACCTCATCCTAACACA<br>GGATTTCCCCTTTTACCTTTATAAACTGCCACTCTCCTATGGGCCATGCCTGTCTCCTCTCTATTCAAAGAC<br>AGTCCTTTGTCCCCTCTTCGAGAGAAATACCTGCCCCCCCGTCCCCCCCCTCCCCTCTTCTCTTTCCCCTT<br>CTCCATCTCCTGTCTTGTCTCTTATTCCTATACTTCGTCCCTCTAGGGCAAATAAAGCTCCTTTGTCCTGAA<br>AACTTGGTCTTGGGGTGTCCTGAGCCGATACCAGTCCCTTCAAGGCTGCAGTTTGAGTTGCGAATGGGAAGA<br>GCCAGGCTTGAAGTGGACGCAGCGCCTCGTCTCCGCACCTCACACAGCACGGAGCCGCTCTAGCCACACTGG<br>GACTCGGTGGCGGCCGGAGTTGCACGTCCTGGCACCTCCTCCCTGAGCAGGTGAGTTCGCCGGTGGAGGAGG<br>GGGTGCAAGGGTCCACGGGACGGACGGACGGGCGGGCGGGCACTGCT	551bp
	inv	CTAAAAACGTTAGATTGAGTTCCTAAAGCTAGCTACCGAGATCCGTTCCCTTATTCACCTCATCCTAACACA<br>GGATTTCCCCTTTTACCTTTATAAACTGCCACTCTCCTATGGGCCATGCCTGTCTCCTCTCTATTCAAAGAC<br>AGTCCTTTGTCCCCTCTTCGAGAGAAATACCTGCCCCCCCGTCCCCCCCCTCCCCTCTTCTCTTTCCCCTT<br>CTCCATCTCCTGTCTTGTCTCTTATTCCTATACTTCGTCCCTCTAGGGCAAATAAAGCTCCTTTGTCCTGAA<br>AACTTGGTCTTGGGGTGTCCTGAGCCGATACCAGTCCCTTCAAGGCTGCAGTTTGAGTTGCGAATGGGAAGA<br>GCCCTGCGCCCCCTGCAGGCCTCGCCTCGTCTCCGCACCTCACACAGCACGGAGCCGCTCTAGCCACACTGG	551bp

		GACTCGGTGGCGGCCGGAGTTGCACGTCCTGGCACCTCCTCCCTGAGCAGGTGAGTTCGCCGGTGGAGGAGG<br>GGGTGCAAGGGTCCACGGGACGGACGGACGGGCGGGCGGGCACTGCT	
Dusp12	wt	ATATAAGCTTATACATACCAGATTTGTCTTTCAGGGTCTGGGTTACCTCACTCAGAATGATTTTTTTTTTTT<br>TGAGTTTCATCCATTTATCTGCAAATTTCATTTTTTAAAAATGAATACCTTATTGTGTGTAAATGTACCACA<br>TTTTCTTTATTCAGTCTTCTGTTGTACATCTAGGTTGTTTCCAATTTCTGGCTATTATGAAGCATCAATAGA<br>CAACATGGTTGGGCAAATGTCTCAAATGGTAGGGAGAGTGGCTTTGCTGGATCTTGAGATAGATAGATTCCC<br>ATCTTCCTGAGGAACCACCAAGCTGATTTCCATAGTGACTGTACAAGTTTGTGGTCCTACCAGCAACGGATG<br>AATGGAGACTTTATTTTTCTGCCTTGGACTTTAGAAGGTTGGGTGGAAAAGCTGCTCTAAATGGAGCTTATT<br>TAGCACACAGCCCCAGCACGGCTCACATCACCTTCCCCAGAAGCAGTCATTTTCCCTATTGCCATGGTCCTC<br>CCACGGCGCTGCTGAGCGGCTGGAGCTATTCCGACACAGGAGTCAGTCACTCTAGCCGGTGACTTGGTGGCG<br>GGCAGCGGCTGCCTGGCTTCCTGGCTCTGCCATGTTGGAAG	617bp
	mut	ATATAAGCTTATACATACCAGATTTGTCTTTCAGGGTCTGGGTTACCTCACTCAGAATGATTTTTTTTTTTT<br>TGAGTTTCATCCATTTATCTGCAAATTTCATTTTTTAAAAATGAATACCTTATTGTGTGTAAATGTACCACA<br>TTTTCTTTATTCAGTCTTCTGTTGTACATCTAGGTTGTTTCCAATTTCTGGCTATTATGAAGCATCAATAGA<br>CAACATGGTTGGGCAAATGTCTCAAATGGTAGGGAGAGTGGCTTTGCTGGATCTTGAGATAGATAGATTCCC<br>ATCTTCCTGAGGAACCACCAAGCTGATTTCCATAGTGACTGTACAAGTTTGTGGTCCTACCAGCAACGGATG<br>AATGGAGACTTTATTTTTCTGCCTTGGACTTTAGAAGGTTGGGTGGAAAAGCTGCTCTAAATGGAGCTTATT<br>TAGCACACAGCCCCAGCACGGCTCACATCACCTACCACATAAACAGTCATTTTCCCTATTGCCATGGTCCTC<br>CCACGGCGCTGCTGAGCGGCTGGAGCTATTCCGACACAGGAGTCAGTCACTCTAGCCGGTGACTTGGTGGCG<br>GGCAGCGGCTGCCTGGCTTCCTGGCTCTGCCATGTTGGAAG	617bp
	inv	ATATAAGCTTATACATACCAGATTTGTCTTTCAGGGTCTGGGTTACCTCACTCAGAATGATTTTTTTTTTTT<br>TGAGTTTCATCCATTTATCTGCAAATTTCATTTTTTAAAAATGAATACCTTATTGTGTGTAAATGTACCACA<br>TTTTCTTTATTCAGTCTTCTGTTGTACATCTAGGTTGTTTCCAATTTCTGGCTATTATGAAGCATCAATAGA<br>CAACATGGTTGGGCAAATGTCTCAAATGGTAGGGAGAGTGGCTTTGCTGGATCTTGAGATAGATAGATTCCC<br>ATCTTCCTGAGGAACCACCAAGCTGATTTCCATAGTGACTGTACAAGTTTGTGGTCCTACCAGCAACGGATG<br>AATGGAGACTTTATTTTTCTGCCTTGGACTTTAGAAGGTTGGGTGGAAAAGCTGCTCTAAATGGAGCTTATT<br>TAGCACACAGCCCCAGCACGGCTCACATCATGACTGCTTCTGGGGAAGGTTTTCCCTATTGCCATGGTCCTC<br>CCACGGCGCTGCTGAGCGGCTGGAGCTATTCCGACACAGGAGTCAGTCACTCTAGCCGGTGACTTGGTGGCG<br>GGCAGCGGCTGCCTGGCTTCCTGGCTCTGCCATGTTGGAAG	617bp
Tab1	wt	GCCAGTCACGCCAAGTGGCCTGCAATTACATTGCCAGCTAACACTACACTCCCATGTTACCGTCCATAAATT<br>TAACTTCTGAGAATTTACAACTACTCGGAATTGCAGTCTTCTCCAAATCTAACTTCAGCTAGTTTGGCATTG<br>GGGTGGCCTCCATTATAAGATCAGGCAGTCAGTCACCCACTACTCTTGTAAACTGAATCATTCGTTCTCTCT<br>TGCTCCCTCCTCGCTAAGCCGCTTTAAATAGGATCTCAGGATCTCCATCGGGGTGAAAAAAAAATCCGGGAG<br>AAAGCACACCCATAAAGACCCCAGGAAACAAGAAAGTCGAAGAAAGTAGGCTGATGGGGTTGGGGGTGGGGT<br>GGGGGCAAGAGCCGCGCTTGCTCAAGGCGCCTCTGCTGCGCCTCCTCCGCTCCGCCGGGGGCGCTGTGGATA<br>AGCTCCGCGGCAGCCCGAGCCGCTCTAGCCCCTCATCCCGCCACTCCGAGAGCCTGGCGCGCCGCGGGGTCC<br>TCCAAGAGGCGGCGCAGAGGAGGAGCCTGCTCCAGAGTGTGAGGA	549bp
	mut	GCCAGTCACGCCAAGTGGCCTGCAATTACATTGCCAGCTAACACTACACTCCCATGTTACCGTCCATAAATT<br>TAACTTCTGAGAATTTACAACTACTCGGAATTGCAGTCTTCTCCAAATCTAACTTCAGCTAGTTTGGCATTG<br>GGGTGGCCTCCATTATAAGATCAGGCAGTCAGTCACCCACTACTCTTGTAAACTGAATCATTCGTTCTCTCT<br>TGCTCCCTCCTCGCTAAGCCGCTTTAAATAGGATCTCAGGATCTCCATCGGGGTGAAAAAAAAATCCGGGAG<br>AAAGCACACCCATAAAGACCCCAGGAAACAAGAAAGTCGAAGAAAGTAGGCTGATGGGGTTGGGGGTGGGGT<br>GGGGGCAAGAGCCGCGCTTGATCACAGCTCCTCTGCTGCGCCTCCTCCGCTCCGCCGGGGGCGCTGTGGATA<br>AGCTCCGCGGCAGCCCGAGCCGCTCTAGCCCCTCATCCCGCCACTCCGAGAGCCTGGCGCGCCGCGGGGTCC<br>TCCAAGAGGCGGCGCAGAGGAGGAGCCTGCTCCAGAGTGTGAGGA	549bp
	inv	GCCAGTCACGCCAAGTGGCCTGCAATTACATTGCCAGCTAACACTACACTCCCATGTTACCGTCCATAAATT<br>TAACTTCTGAGAATTTACAACTACTCGGAATTGCAGTCTTCTCCAAATCTAACTTCAGCTAGTTTGGCATTG<br>GGGTGGCCTCCATTATAAGATCAGGCAGTCAGTCACCCACTACTCTTGTAAACTGAATCATTCGTTCTCTCT<br>TGCTCCCTCCTCGCTAAGCCGCTTTAAATAGGATCTCAGGATCTCCATCGGGGTGAAAAAAAAATCCGGGAG<br>AAAGCACACCCATAAAGACCCCAGGAAACAAGAAAGTCGAAGAAAGTAGGCTGATGGGGTTGGGGGTGGGGT<br>GGGGGCAAGAGCCGCGCAGAGGCGCCTTGAGCAAGCTGCGCCTCCTCCGCTCCGCCGGGGGCGCTGTGGATA<br>AGCTCCGCGGCAGCCCGAGCCGCTCTAGCCCCTCATCCCGCCACTCCGAGAGCCTGGCGCGCCGCGGGGTCC<br>TCCAAGAGGCGGCGCAGAGGAGGAGCCTGCTCCAGAGTGTGAGGA	549bp
Mrpl23	wt	GGCCATGCAATGTTGGGGTGGGGAAACAGGATAGTAGTTGCCCTTTGTAGAATCCACCAGCCAGCAGTGCAG<br>AGATGCGGGACTGCTCATGGCAAAGTGTGGTGCCCTTGTGGGTGGGAAAGCATGTACTGTAGGGTGCTTACA<br>GGTTGAAGAACAGGGAGGTACACTATACACCGAGAGGTATGCAGGATTCTGTGTGGTGCTGGGATGGGGCAA<br>ATGGGTACAGAGTGTATGCCGGCTTTTTGCGGGGCCGGGTGAGGGGCGGGGCTGTTCACTGTTTTTATGGCT<br>GAGAGGCGGGGTGCGCTGTGCCGGGGGCGGTGCGCAAGCAGCTTACGGTGCACGGGTGGGGGAGGGGCTTTT<br>CGGGTGCGGGGTTGGGGGTGGGGTGGGGTTTGCGGGACACCGAAAGTGCGGGGCCCTGCGGTGCAGGGAGAG<br>GGTAGCGCGGCCTCCGGAAGCGAGGGGGCGCTGTGCCAGCGCGGGCGCTGTGCGGGGCGGGTGTCGCTCCTT<br>CCTGACCAGGAGGTGCGGCTGCCATGGCGCGGAATGTGTTGTGAG	549bp
	mut	GGCCATGCAATGTTGGGGTGGGGAAACAGGATAGTAGTTGCCCTTTGTAGAATCCACCAGCCAGCAGTGCAG<br>AGATGCGGGACTGCTCATGGCAAAGTGTGGTGCCCTTGTGGGTGGGAAAGCATGTACTGTAGGGTGCTTACA<br>GGTTGAAGAACAGGGAGGTACACTATACACCGAGAGGTATGCAGGATTCTGTGTGGTGCTGGGATGGGGCAA<br>ATGGGTACAGAGTGTATGCCGGCTTTTTGCGGGGCCGGGTGAGGGGCGGGGCTGTTCACTGTTTTTATGGCT<br>GAGAGGCGGGGTGCGCTGTGCCGGGGGCGGTGCGCAAGCAGCTTACGGTGCACGGGTGGGGGAGGGGCTTTT<br>CGGGTGCGGGGTTGGGGGTGGGGTGGGGTTTGCGGGACACCGAAAGTGCGGGGCCCTGCGGTGCAGGGAGAG<br>GGTAGCGCGGCCTCCGGAAGCGCAGGACCGCTGTGCCAGCGCGGGCGCTGTGCGGGGCGGGTGTCGCTCCTT<br>CCTGACCAGGAGGTGCGGCTGCCATGGCGCGGAATGTGTTGTGAG	549bp
	inv	GGCCATGCAATGTTGGGGTGGGGAAACAGGATAGTAGTTGCCCTTTGTAGAATCCACCAGCCAGCAGTGCAG<br>AGATGCGGGACTGCTCATGGCAAAGTGTGGTGCCCTTGTGGGTGGGAAAGCATGTACTGTAGGGTGCTTACA<br>GGTTGAAGAACAGGGAGGTACACTATACACCGAGAGGTATGCAGGATTCTGTGTGGTGCTGGGATGGGGCAA<br>ATGGGTACAGAGTGTATGCCGGCTTTTTGCGGGGCCGGGTGAGGGGCGGGGCTGTTCACTGTTTTTATGGCT<br>GAGAGGCGGGGTGCGCTGTGCCGGGGGCGGTGCGCAAGCAGCTTACGGTGCACGGGTGGGGGAGGGGCTTTT<br>CGGGTGCGGGGTTGGGGGTGGGGTGGGGTTTGCGGGACACCGAAAGTGCGGGGCCCTGCGGTGCAGGGAGAG<br>GGTAGCGCGGCCTCCAGCGCCCCCTCGCTTCCGTGCCAGCGCGGGCGCTGTGCGGGGCGGGTGTCGCTCCTT<br>CCTGACCAGGAGGTGCGGCTGCCATGGCGCGGAATGTGTTGTGAG	549bp
Mrps2	wt	CGGGGCGGTGGTAGGGCATGCCTTCAATCTCAGCTCTCCAGAGGCAGAGGCGGGTTGCTCTCTGAGTTCAAG<br>CCAACCTGGTCTACATAGCGAATACCATGACAGTCAAGGCTCAAGGCTACGGAGAAACCGGGTCTCAGGAAA<br>AACAAAACAAAACAAAACAAAACAAAACAAAACAAAACAAAAACAAAAACAAAAACAAAAACAACAAAAAAA	573bp

		CAGCAACCCTAAAAACAACCTCTAAAAACCAATTTGAACAAGAGGGTCGCCTCCTCCAGAGGATGCTGTAAT<br>TTTATCGAGAGGAACGAGCTGTGGGTGAGAGGTCGCTCATTCTAGCTCAGCCCCACGTCCCAGCTGAGTGCA<br>GACCACTGGTCACAGTGCCAACTCGCCCATTTACAAGGATGCGGGCGCAGCGAGCGGTGCACAGCGGAGAGG<br>GTCCTCCGGGCCAGCAAGCGGCGCTGCGACCCCGCGCGCTCAGAGGAGGAGGCGCTCTGCCGGGCAGGAAGA<br>GTCTCTGCAGGATCTCTGTCCACGCGCCCGCCATGGCGCCCGCTCCGGCCGTGCTGACCCGGCTGCTGT	
	mut	CGGGGCGGTGGTAGGGCATGCCTTCAATCTCAGCTCTCCAGAGGCAGAGGCGGGTTGCTCTCTGAGTTCAAG<br>CCAACCTGGTCTACATAGCGAATACCATGACAGTCAAGGCTCAAGGCTACGGAGAAACCGGGTCTCAGGAAA<br>AACAAAACAAAACAAAACAAAACAAAACAAAACAAAACAAAAACAAAAACAAAAACAAAAACAACAAAAAAA<br>CAGCAACCCTAAAAACAACCTCTAAAAACCAATTTGAACAAGAGGGTCGCCTCCTCCAGAGGATGCTGTAAT<br>TTTATCGAGAGGAACGAGCTGTGGGTGAGAGGTCGCTCATTCTAGCTCAGCCCCACGTCCCAGCTGAGTGCA<br>GACCACTGGTCACAGTGCCAACTCGCCCATTTACAAGGATGCGGGCGCAGCGAGCGGTGCACAGCGGAGAGG<br>GTCCTCCGGGCTAGAAATCGACGCTGCGACCCCGCGCGCTCAGAGGAGGAGGCGCTCTGCCGGGCAGGAAGA<br>GTCTCTGCAGGATCTCTGTCCACGCGCCCGCCATGGCGCCCGCTCCGGCCGTGCTGACCCGGCTGCTGT	573bp
	inv	CGGGGCGGTGGTAGGGCATGCCTTCAATCTCAGCTCTCCAGAGGCAGAGGCGGGTTGCTCTCTGAGTTCAAG<br>CCAACCTGGTCTACATAGCGAATACCATGACAGTCAAGGCTCAAGGCTACGGAGAAACCGGGTCTCAGGAAA<br>AACAAAACAAAACAAAACAAAACAAAACAAAACAAAACAAAAACAAAAACAAAAACAAAAACAACAAAAAAA<br>CAGCAACCCTAAAAACAACCTCTAAAAACCAATTTGAACAAGAGGGTCGCCTCCTCCAGAGGATGCTGTAAT<br>TTTATCGAGAGGAACGAGCTGTGGGTGAGAGGTCGCTCATTCTAGCTCAGCCCCACGTCCCAGCTGAGTGCA<br>GACCACTGGTCACAGTGCCAACTCGCCCATTTACAAGGATGCGGGCGCAGCGAGCGGTGCACAGCGGAGAGG<br>GTCCTCCCAGCGCCGCTTGCTGGCCCCGACCCCGCGCGCTCAGAGGAGGAGGCGCTCTGCCGGGCAGGAAGA<br>GTCTCTGCAGGATCTCTGTCCACGCGCCCGCCATGGCGCCCGCTCCGGCCGTGCTGACCCGGCTGCTGT	573bp
Ttc5	wt	GTTCTCTTCCAAATACCACCTGACATGCATACTTTCATAGTCGGCCTTGCTTTCTAAGAGTGATGTAAAAAA<br>GGACAAGAACATACAAAACAAAACAAAAAAAAATGAATGGAATTAATTTAAGAACATTTTGATTCCAGAATTT<br>GGGAAGGGAATTATAAAACTAGGAAAATTATTTATGAAACCGAAAGTGACCACAAACTCAGCAGGTGTTTGT<br>TATCCCAGTTTGAGGGTCTGTGGCAAAAGTTCAGCCTGGACTACATAGCCACGTTGAGGCCAATCTGATCTA<br>CGTGGTGGGTGAGATTCTGTCGCAATAAAAAGAAGCAAAACAAAACCCCAAAGCAGTTCTAGGGCATCTTTG<br>AGACACCAAGTTCTGGATTAAGTTGGTTTGTAGCTGTGTCTCTGAGGCGCAGATGGGCCTCCGGAGCCCCAG<br>GGGTCGCTGTGGTTTCAGCGTAGGCATGGCTGAGTCTCTGTGGTTGAAAAGTTGGGGGAGGAGACGCTGAGA<br>GCGGCTGGGATGATGGCTGATGAAGAGGAAGAAGCGAAGC	544bp
	mut	GTTCTCTTCCAAATACCACCTGACATGCATACTTTCATAGTCGGCCTTGCTTTCTAAGAGTGATGTAAAAAA<br>GGACAAGAACATACAAAACAAAACAAAAAAAAATGAATGGAATTAATTTAAGAACATTTTGATTCCAGAATTT<br>GGGAAGGGAATTATAAAACTAGGAAAATTATTTATGAAACCGAAAGTGACCACAAACTCAGCAGGTGTTTGT<br>TATCCCAGTTTGAGGGTCTGTGGCAAAAGTTCAGCCTGGACTACATAGCCACGTTGAGGCCAATCTGATCTA<br>CGTGGTGGGTGAGATTCTGTCGCAATAAAAAGAAGCAAAACAAAACCCCAAAGCAGTTCTAGGGCATCTTTG<br>AGACACCAAGTTCTGGATTAAGTTGGTTTGTAGCTGTGTCTCTGAGGCGCAGATGGGCCTCCGGAGTCCCCA<br>GGATCGCTGTGGTTTCAGCGTAGGCATGGCTGAGTCTCTGTGGTTGAAAAGTTGGGGGAGGAGACGCTGAGA<br>GCGGCTGGGATGATGGCTGATGAAGAGGAAGAAGCGAAGC	544bp
	inv	GTTCTCTTCCAAATACCACCTGACATGCATACTTTCATAGTCGGCCTTGCTTTCTAAGAGTGATGTAAAAAA<br>GGACAAGAACATACAAAACAAAACAAAAAAAAATGAATGGAATTAATTTAAGAACATTTTGATTCCAGAATTT<br>GGGAAGGGAATTATAAAACTAGGAAAATTATTTATGAAACCGAAAGTGACCACAAACTCAGCAGGTGTTTGT<br>TATCCCAGTTTGAGGGTCTGTGGCAAAAGTTCAGCCTGGACTACATAGCCACGTTGAGGCCAATCTGATCTA<br>CGTGGTGGGTGAGATTCTGTCGCAATAAAAAGAAGCAAAACAAAACCCCAAAGCAGTTCTAGGGCATCTTTG<br>AGACACCAAGTTCTGGATTAAGTTGGTTTGTAGCTGTGTCTCTGAGGCGCAGATGGGCCTCCCAGCGACCCC<br>TGGGGCTCCTGGTTTCAGCGTAGGCATGGCTGAGTCTCTGTGGTTGAAAAGTTGGGGGAGGAGACGCTGAGA<br>GCGGCTGGGATGATGGCTGATGAAGAGGAAGAAGCGAAGC	544bp
Actn4	wt	ATTTAAGACTGGTCTTCCTGTGTAACCCAAGCTCCAACATGTTATCTGTCGTTCAGTCTCTGCGGCCTGAGA<br>ACAGAGATCCCACGTTCCATCGTGCAAGGTTTATAGTATATAGTTTCGAAAGGGTGGGGACTTTTCCTGACT<br>TCTTTAATGCCAAGGCAGAAAACACGGTCTCACACATAGCAGGCAGCCAAATAAATCTTGATTATGTCTGAA<br>TGGCTGAATGGGAACACATTCCACCCCGAAACAGGTTAAAGCCGGATGAGGAGCCCCGCCTTATGAATCATT<br>CATGACTTGGGCCCCGCCCACACGGGGTGACGCGCGCGTTCCCGCTAGAAGCGCCTGGGCTCCCACGCTGGG<br>CGGGCAGCCGGAGGTGCGTTCTGCGCCGGCGCCGAGAGCCCGGGGCCTCCTCAGGTGCGCGCGCGCGGGAGG<br>GCGGCGGCGGCAGCGCAGTGCGCAGGCGCGGCAAGTGAACTGCGCAGGCGTCAGTCTTGGGCGGGCTGAAAG<br>CAGCTGAAGCGGCGGCCGAGTCGGAGCCTCCGGCACAGGGGCGGGA	550bp
Psmd4	wt	AGTAGTGTATGCACTCATGAGTGTCTGTTATGTGTTCGAGGCAGTCTCACTACGTAGTAGCTATGTTGCCCA<br>CGTTGGCCTGGAAATTGCAGTTCAGATCTTAGCATGAGTGTGCCCCTTGTAAACAGCACTGGGAGGTGAAGC<br>AATAGAGTTTATTAGCCTGAGCTCCACAGCGAATTGCTGTCATCCTTTTTCAAACCGTAACAACAAAAACAA<br>ATCAAAAACCCAAATATACAAAAGAAAGAGAGCAGAAGAGGGGGTAAATTACAGCTCTCCTGCCTCACACTG<br>GAGTGTTGCTGCCAGGCGAGTGCAGAGAGCTTACAGCTGTTCCGGAAGCTGCTGCGAAGGTAGCCAACTCTA<br>TTGGATAACCAACCTTTCCATTTCTGTGGCGCCTACAAGTCCCAGAACACACCGCGGGGCGCGGAGGCGGGG<br>CAGCATGAAGACTGAGTTCTTTGATTGGTGATGAGCACCAGGCCAATCGGAGGTGCTGTTGTTTGTCAGTCC<br>CGGAGCCGGTCCGCAGAGGGAAGGAGGCAAGATGGTGTTGGAGA	548bp

Supplementary Notes

Supplementary Note 1

Most genes regulated after $CTCF^{AID}$ and $RAD21^{AID}$ depletion did not pass statistical thresholds. The following evidence supports that weak transcription changes observed in our analyses are non-random and reflect genuine perturbation-induced changes:

1. CTCF-regulated genes identified in EU-seq data are consistently regulated in independently published RNA-seq data in the same cell line (**Extended Data Fig. 4e, f**).
2. Most $RAD21^{down}$ identified in initial experiments show consistent downregulation in additional replicates (**Extended Data Fig. 2d**).
3. Genes down-regulated by $CTCF^{AID}$ and $RAD21^{AID}$ depletion strongly differ in their regulation by A-485 in mESC (**Fig. 1,2**).
4. Like mESC, $RAD21^{AID}$ depletion in NPC causes preferential downregulation of CBP/p300-dependent genes (**Fig. 1**).
5. Among $CTCF^{down}$ genes, those not downregulated by A-485 are strongly bound by CTCF at their promoters, while those downregulated by A-485 show no strong CTCF binding (**Fig. 3d-e**).
6. Genes regulated by CTCF and RAD21 show notable differences in cell-type specificity (**Fig. 4a**). Within CTCF-downregulated genes, those requiring CTCF-anchored loops and those not requiring loops show opposite cell-type-specific expression patterns.
7. $CTCF^{down}$ genes exhibit strong polarity in CTCF binding at their promoters (**Fig. 4e**).
8. $CTCF^{down}$A-485ND genes exhibit strong positional binding of CTCF within a narrow, TSS upstream window (**Fig. 4g**).
9. The loop anchoring-defective $CTCF^{YF\text{-}AA}$ mutant activates promoters of several $CTCF^{down}$ genes in reporter assays (**Fig. 5a**).
10. Overexpression of BORIS selectively restores transcription of a subset of $CTCF^{down}$ genes bound by CTCF at their promoters (**Fig. 5b**).
11. $CTCF^{AID}$ depletion-induced transcription changes in EU-seq strongly correlate with CTCF-dependent promoter chromatin accessibility in ATAC-seq, analyzed using an independently generated ^{dTAG}CTCF depletion cell line (**Fig. 6a-c**).
12. $CTCF^{AID}$ depletion-induced transcription changes in EU-seq strongly correlate with CTCF-dependent recruitment of Pol II in ChIP-seq, again measured in an independently generated ^{dTAG}CTCF cell line (**Fig. 6d-e**).

These results in different cell types (mESC, NPC), independently generated cell lines ($CTCF^{AID}$, ^{dTAG}CTCF, $CTCF^{YF\text{-}AA}$, $^{bTAG_\Delta N}$CTCF / $^{dTAG}CTCF^{\Delta C}$), and different molecular assays (EU-seq, RNA-seq, ATAC-seq, ChIP-seq, and plasmid reporters) support the presented conclusions.

Supplementary Note 2

It is suggested that the removal of cohesin results in gene downregulation through increased Polycomb-mediated repression[4]. To investigate whether gene downregulation after $RAD21^{AID}$ depletion results from reduced enhancer targeting or increased Polycomb interaction, we examined Polycomb-catalyzed H3K27me3. H3K27me3 is more frequently enriched in low (TPM 5-15) expressed as compared to high (TPM >15) expressed genes, and genes regulated by A-485, CTCF, and RAD21 show slightly more H3K27me3 enrichment than not regulated genes (**Extended Data Fig. 2f**). Nevertheless, at the used expression threshold (TPM >15), only ~15% of $RAD21^{down}$ genes are marked by H3K27me3, which is comparable to H3K27me3-positive A-485^{down} genes. Lack of

H3K27me3 in majority of RAD21^{down} genes indicates that their downregulation is likely caused by other mechanisms, likely loss of enhancer interactions.

Supplementary Note 3

Why do cohesin and CTCF removal only affect a subset of candidate enhancer target genes? One explanation is that some E–P interactions can occur independently of cohesin[5,6], and potentially through alternative mechanisms involving factors like LDB1, YY1, Mediator, or Pol II[7-12]. Some of these factors, such as Pol II and Mediator, bind broadly across active promoters and enhancers, and it remains unclear how such broadly binding factors could promote interactions between specific enhancer-gene pairs.

Inspired by Paul Nurse´s call for biologists to generate both new data and ideas[13], we propose an alternative model to stimulate discussion on this topic. We posit that architectural proteins mediate some E-P interactions, but this is not a universal requirement for all enhancers. Instead, affinities between the proteins binding in enhancers and promoters, such as transcription factors, chromatin remodelers, and coactivators, may be sufficient to mediate shorter-range, transient E-P interactions. Specificity of such interactions could simply be afforded by the linear proximity between enhancers and promoters. This idea is supported by recent findings that transcription factors and chromatin remodelers can independently drive higher-order chromatin organization in yeast without specific architectural proteins[14]. However, in the absence of a dedicated looping mechanism, the likelihood of such affinity-driven E-P interactions would decrease with increased distance. Cohesin-dependent loop extrusion can overcome this limitation, allowing enhancers to act at much longer genomic distances. This model can explain why cohesin is not universally required by enhancers, and why the synthetic insertion of an enhancer near non-enhancer-regulated genes in the native context can effectively boost the transcription of proximal genes[15]. This can also explain the differences in the cohesin requirement for proximal and distal enhancers in synthetic systems[16,17]. Importantly, this concept could also explain the striking genome-scale association between the distance and strength of CBP/p300-dependent enhancers and their dependency on CBP/p300 activity in the native context[18].

Supplementary Note 4

Since most strongly downregulated genes in CTCF-depleted cells depend on its non-architectural functions, why has CTCF's role as an activator been largely overlooked in current models? For over a decade, studies have noted that genes downregulated after CTCF depletion are enriched for CTCF binding at their promoters[1,19-22]. However, for several reasons, its role as a transcription activator has been largely overlooked. Early studies identified CTCF as both an activator and repressor[23-26], but these conclusions were based on analyses of a few promoters in reporter assays, leaving their relevance to in vivo gene regulation unclear. High-throughput promoter reporter assays confirmed CTCF's repressor function but failed to validate its activator role[27]. Acute CTCF depletion further showed that promoter-bound CTCF prevents antisense transcription without noticeably affecting sense-strand transcription[28]. After its discovery as a genome organizer, its gene regulatory function was reinterpreted exclusively through loop anchoring, dismissing its activator function[29,30]. Supporting this model, genome-wide analyses revealed that promoter-bound CTCF facilitates E-P interactions[31-34], influences enhancer target selection[31-34], and anchors cohesin loops to activate protocadherin genes[35].

As a result, CTCF's role as a 3D genome organizer has dominated the field, with its impact on gene regulation viewed through this lens[29]. By identifying cohesin's preference for CBP/p300-dependent enhancer-driven gene activation and distinguishing CTCF's cohesin-dependent and -independent functions, our findings reconcile historical and contemporary models of CTCF's role in gene regulation.

Supplementary Methods

Data analysis

Processing of ChIP-seq data

Adaptor trimming was performed as described in the RNA-seq section. Read sequences were aligned to the mm10 mouse or hg19 human genome using bwa meme (version 1.0.4) [36] with the soft clipping option for supplementary alignments. Duplicated reads were annotated and removed using Picard toolkit tools (version 2.9.1, "Picard Toolkit." 2019. Broad Institute, GitHub Repository. https://broadinstitute.github.io/picard/; Broad Institute). For the paired-end reads, only the properly aligned read pairs were retained, using samtools. Peak calling of CTCF, RAD21, H2BK20ac, and H3K27ac, TBP, and H3K4me3 ChIP-seq was performed using LanceOtron with the default model (wide-and-deep_jan-2021)[37]. Peak calling of H3K27me3 was performed using epic2 (version 0.0.47) [38]. In the H2BK20ac and H3K27ac datasets, the peaks proximal within 2kb are merged using Bedtools[39]. Poorly enriched peaks of maximum peak height < 8 reads mapped per million (rpm) were omitted. Peak heights were calculated using bamCompare[40] with the following parameters (centerReads, minMappingQuality 10, bin size 20bp, smoothing length 400bp, extension of reads to 200bp, rpm normalization, and input rpm value subtracted). The peak summit was defined as the center of the 20bp bin at maximum height in each peak region. To identify the precise peak summit position of CTCF, CTCF ChIP-exo peaks were determined using GEM (version 3.4) [41] with the following parameters (minimus k-mers 6, maximum k-mers 13, smoothing 3bp, maximum read count on a base position 20). The CTCF ChIP-seq peaks with summit positions within 200bp from the center of the ChIP-exo peak region were retained and used for further analysis. CTCF peaks were categorized into quartiles Q4, Q3, Q2, and Q1 based on the ChIP-seq peak height, from highest to lowest. The CTCF peak summit positions were defined by the center of ChIP-Exo peak regions. EPDnew [42] was used to determine the precise TSS position as follows; If the EPD TSS position was located within 200b from the GENCODE TSS position of the corresponding gene, the EPD TSS position was used, otherwise GENCODE TSS position was used. The distance between ChIP-seq peak and the TSS indicates the distance between the peak summit and the TSS. For the calculation of Pol II ChIP-seq changes after ^{dTAG}CTCF degradation, we assumed that Pol II binding remains unaffected at gene body regions of the genes that are not changed (NC) in EU-seq analysis of CTCF^{AID}-depleted cells. The scaling factors were calculated by DESeq2 default normalization methods using the Pol II ChIP read counts at gene body regions of NC genes, defined by EU-seq transcript fold-change after CTCF^{AID} depletion. The aggregate ChIP-seq profiles were plotted using deepTools2 (version 3.5.2) [40]. For gene track visualization, IGV[43] (version2.16) was used. For the visualization of ChIP-seq and ATAC-seq profiles on gene track, we used 1bp binned, input-subtracted values without any smoothing (CTCF, TBP, Pol II, and ATAC-seq), or 20bp binned, input-subtracted values with smoothing using proximal 400bp (H3K4me3).

Defining enhancer strength

Enhancer regions were defined as overlapping regions of H2BK20ac and H3K27ac peaks. To assess the enhancer strength, H2BK20ac reads were counted within these regions. Reads mapped near TSS (+/- 500bp regions) were excluded to avoid the potential contribution of promoter acetylation. The read count was normalized by using reads per million (rpm), and input rpm values were subtracted

at the corresponding regions. Peaks with H2BK20ac > 1 rpm were retained and used to define enhancer peaks. These enhancers were then ranked by their H2BK20ac rpm values, and the top 10, 25, 50, and 75% enhancers were classified based on their H2BK20ac enrichment within the identified enhancers.

Processing of ATAC-seq data
Adaptor trimming was performed as described in the RNA-seq section. Paired-end read sequences were aligned to mm10 genome using BWA meme (version 1.0.4)[36] with the soft clipping option for supplementary alignments. Duplicated read pairs were removed using Picard-tools (version 2.9.1, "Picard Toolkit." 2019. Broad Institute, GitHub Repository. https://broadinstitute.github.io/picard/; Broad Institute). We filtered out low-quality reads with a MAPQ score of less than 10 and non-primary alignments and retained only the properly aligned read pairs using samtools. Peak regions were called from the merged alignment files of both control and dTAG-13-treated samples using LanceOtron and its default model (wide-and-deep_jan-2021)[44]. Peak height was calculated using bamCoverage with the following parameters (centerReads, bin size 20bp, smooth length 400bp, extend reads 200bp, rpm normalization). Poorly-enriched peaks of peak summit height < 8 rpm were filtered out. For the calculation of ATAC accessibility changes, ATAC reads that were mapped in the summit +/- 100bp regions were counted. After filtering out the low-mapped regions (average reads ≤ 20 between replicates in both control and treatment conditions), log2 fold-change (FC) was calculated with the default scaling method of DESeq2[45], using the median of relative abundance.

Analysis of CTCF ChIP-seq data from different human and mouse cell lines and tissues
The publicly available human and mouse CTCF ChIP-seq data were downloaded from the GEO repository, and peaks were called as described in the processing ChIP-seq data section. The criteria for selecting datasets were the following: 1. Corresponding input data is registered in the same series, 2. Peak number > 20,000. One dataset was chosen for each tissue /cell line. As a result, 49 mouse and 65 human tissues/cell line CTCF peak data were used for the analysis. In each dataset, the distance between the TSS of "protein_coding" or "lincRNA" transcripts and the nearest peak summit was calculated. If the CTCF peak summit position is within –200bp to +50bp of TSS, the corresponding gene was defined as CTCF peak (+). Each CTCF peak was ranked by the peak height from highest to lowest, and the top 20,000 peaks in each dataset were used for comparison. P-values were calculated using the Kolmogorov-Smirnov test, and the Benjamini & Hochberg method was used for multiple comparison correction.

CTCF motif analysis
Enriched motifs were detected by comparing the sequences of +/- 50bp region from CTCF peak summits with their dinucleotide shuffled sequences. STREME (version 5.5.4) [46] was used for motif elicitation by the following parameters (minimum motif length 6, maximum motif length 30, differential motif detection mode) and compared with CTCF (MA0139.1**)** from JASPAR database (https://jaspar.genereg.net). To summarize the motif occurrences and their orientations, the output motifs from STREME were scanned using the +/- 100bp region from CTCF peak summits using fimo (version 5.5.4) [47]. The motif orientation of promoter-proximal CTCF peaks was determined with respect to the orientation of the corresponding gene.

Gene expression profiles of various tissues in mouse
Cell type-specificity of genes was classified using the FANTOM5 CAGE dataset [48,49]. In the mouse CAGE dataset, we excluded data from "whole," "lactating," and "pregnant" stages. The resultant 151 mouse tissue expression profiles (44 tissues from 11 developing stages) were converted into a binary expression matrix using a gene expression threshold of TPM ≥ 2.

Analysis of common essential genes

The processed CRISPR gene effect datasets and common essential gene list were downloaded from DepMap portal (version 23Q2) [50]. Human orthologues of mouse genes were determined by using the Ensembl Orthologue dataset [51] with the following criteria. 1. Homology type is "ortholog_one2one", or 2. Homology type is either "ortholog_one2many" or "ortholog_many2one" and the confidence score is 1.

Supplementary references

1 Hsieh, T. S. *et al.* Enhancer-promoter interactions and transcription are largely maintained upon acute loss of CTCF, cohesin, WAPL or YY1. *Nat Genet* **54**, 1919-1932, doi:10.1038/s41588-022-01223-8 (2022).

2 Vos, E. S. M. *et al.* Interplay between CTCF boundaries and a super enhancer controls cohesin extrusion trajectories and gene expression. *Mol Cell* **81**, 3082-3095 e3086, doi:10.1016/j.molcel.2021.06.008 (2021).

3 Nishana, M. *et al.* Defining the relative and combined contribution of CTCF and CTCFL to genomic regulation. *Genome Biol* **21**, 108, doi:10.1186/s13059-020-02024-0 (2020).

4 Rhodes, J. D. P. *et al.* Cohesin Disrupts Polycomb-Dependent Chromosome Interactions in Embryonic Stem Cells. *Cell Rep* **30**, 820-835 e810, doi:10.1016/j.celrep.2019.12.057 (2020).

5 Thiecke, M. J. *et al.* Cohesin-Dependent and -Independent Mechanisms Mediate Chromosomal Contacts between Promoters and Enhancers. *Cell Rep* **32**, 107929, doi:10.1016/j.celrep.2020.107929 (2020).

6 Goel, V. Y., Huseyin, M. K. & Hansen, A. S. Region Capture Micro-C reveals coalescence of enhancers and promoters into nested microcompartments. *Nat Genet* **55**, 1048-1056, doi:10.1038/s41588-023-01391-1 (2023).

7 Liu, T. *et al.* Matrin3 mediates differentiation through stabilizing chromatin loop-domain interactions and YY1 mediated enhancer-promoter interactions. *Nat Commun* **15**, 1274, doi:10.1038/s41467-024-45386-w (2024).

8 Aboreden, N. G. *et al.* LDB1 establishes multi-enhancer networks to regulate gene expression. *bioRxiv*, 2024.2008.2023.609430, doi:10.1101/2024.08.23.609430 (2024).

9 Lam, J. C. *et al.* YY1-controlled regulatory connectivity and transcription are influenced by the cell cycle. *Nat Genet* **56**, 1938-1952, doi:10.1038/s41588-024-01871-y (2024).

10 Ramasamy, S. *et al.* The Mediator complex regulates enhancer-promoter interactions. *Nat Struct Mol Biol* **30**, 991-1000, doi:10.1038/s41594-023-01027-2 (2023).

11 Barshad, G. *et al.* RNA polymerase II dynamics shape enhancer-promoter interactions. *Nat Genet* **55**, 1370-1380, doi:10.1038/s41588-023-01442-7 (2023).

12 Jerković, I. *et al.* A Scaffolding Element Rewires Local 3D Chromatin Architecture During Differentiation. *bioRxiv*, 2024.2005.2023.595561, doi:10.1101/2024.05.23.595561 (2024).

13 Nurse, P. Biology must generate ideas as well as data. *Nature* **597**, 305, doi:10.1038/d41586-021-02480-z (2021).

14 Oberbeckmann, E., Quililan, K., Cramer, P. & Oudelaar, A. M. In vitro reconstitution of chromatin domains shows a role for nucleosome positioning in 3D genome organization. *Nat Genet*, doi:10.1038/s41588-023-01649-8 (2024).

15 Narita, T. *et al.* Acetylation of histone H2B marks active enhancers and predicts CBP/p300 target genes. *Nat Genet* **55**, 679-692, doi:10.1038/s41588-023-01348-4 (2023).

16 Kane, L. *et al.* Cohesin is required for long-range enhancer action at the Shh locus. *Nat Struct Mol Biol* **29**, 891-897, doi:10.1038/s41594-022-00821-8 (2022).

17 Rinzema, N. J. *et al.* Building regulatory landscapes reveals that an enhancer can recruit cohesin to create contact domains, engage CTCF sites and activate distant genes. *Nat Struct Mol Biol* **29**, 563-574, doi:10.1038/s41594-022-00787-7 (2022).
18 Narita, T. *et al.* The logic of native enhancer-promoter compatibility and cell-type-specific gene expression variation. *bioRxiv*, 2022.2007.2018.500456, doi:10.1101/2022.07.18.500456 (2022).
19 Soshnikova, N., Montavon, T., Leleu, M., Galjart, N. & Duboule, D. Functional analysis of CTCF during mammalian limb development. *Dev Cell* **19**, 819-830, doi:10.1016/j.devcel.2010.11.009 (2010).
20 Zuin, J. *et al.* Cohesin and CTCF differentially affect chromatin architecture and gene expression in human cells. *Proc Natl Acad Sci U S A* **111**, 996-1001, doi:10.1073/pnas.1317788111 (2014).
21 Nora, E. P. *et al.* Targeted Degradation of CTCF Decouples Local Insulation of Chromosome Domains from Genomic Compartmentalization. *Cell* **169**, 930-944 e922, doi:10.1016/j.cell.2017.05.004 (2017).
22 Chervova, A., Festuccia, N., Altamirano-Pacheco, L., Dubois, A. & Navarro, P. A gene subset requires CTCF bookmarking during the fast post-mitotic reactivation of mouse ES cells. *EMBO Rep* **24**, e56075, doi:10.15252/embr.202256075 (2023).
23 Kohne, A. C., Baniahmad, A. & Renkawitz, R. NeP1. A ubiquitous transcription factor synergizes with v-ERBA in transcriptional silencing. *J Mol Biol* **232**, 747-755, doi:10.1006/jmbi.1993.1428 (1993).
24 Klenova, E. M. *et al.* CTCF, a conserved nuclear factor required for optimal transcriptional activity of the chicken c-myc gene, is an 11-Zn-finger protein differentially expressed in multiple forms. *Mol Cell Biol* **13**, 7612-7624, doi:10.1128/mcb.13.12.7612-7624.1993 (1993).
25 Filippova, G. N. *et al.* An exceptionally conserved transcriptional repressor, CTCF, employs different combinations of zinc fingers to bind diverged promoter sequences of avian and mammalian c-myc oncogenes. *Mol Cell Biol* **16**, 2802-2813, doi:10.1128/MCB.16.6.2802 (1996).
26 Vostrov, A. A. & Quitschke, W. W. The zinc finger protein CTCF binds to the APBbeta domain of the amyloid beta-protein precursor promoter. Evidence for a role in transcriptional activation. *J Biol Chem* **272**, 33353-33359, doi:10.1074/jbc.272.52.33353 (1997).
27 Duttke, S. H. *et al.* Position-dependent function of human sequence-specific transcription factors. *Nature* **631**, 891-898, doi:10.1038/s41586-024-07662-z (2024).
28 Luan, J. *et al.* CTCF blocks antisense transcription initiation at divergent promoters. *Nat Struct Mol Biol* **29**, 1136-1144, doi:10.1038/s41594-022-00855-y (2022).
29 de Wit, E. & Nora, E. P. New insights into genome folding by loop extrusion from inducible degron technologies. *Nat Rev Genet* **24**, 73-85, doi:10.1038/s41576-022-00530-4 (2023).
30 Phillips, J. E. & Corces, V. G. CTCF: master weaver of the genome. *Cell* **137**, 1194-1211, doi:10.1016/j.cell.2009.06.001 (2009).
31 Guo, Y. *et al.* CTCF/cohesin-mediated DNA looping is required for protocadherin alpha promoter choice. *Proc Natl Acad Sci U S A* **109**, 21081-21086, doi:10.1073/pnas.1219280110 (2012).
32 Tang, Z. *et al.* CTCF-Mediated Human 3D Genome Architecture Reveals Chromatin Topology for Transcription. *Cell* **163**, 1611-1627, doi:10.1016/j.cell.2015.11.024 (2015).
33 Kubo, N. *et al.* Promoter-proximal CTCF binding promotes distal enhancer-dependent gene activation. *Nat Struct Mol Biol* **28**, 152-161, doi:10.1038/s41594-020-00539-5 (2021).

34 Oh, S. *et al.* Enhancer release and retargeting activates disease-susceptibility genes. *Nature* **595**, 735-740, doi:10.1038/s41586-021-03577-1 (2021).

35 Guo, Y. *et al.* CRISPR Inversion of CTCF Sites Alters Genome Topology and Enhancer/Promoter Function. *Cell* **162**, 900-910, doi:10.1016/j.cell.2015.07.038 (2015).

36 Jung, Y. & Han, D. BWA-MEME: BWA-MEM emulated with a machine learning approach. *Bioinformatics* **38**, 2404-2413, doi:10.1093/bioinformatics/btac137 (2022).

37 Hentges, L. D., Sergeant, M. J., Downes, D. J., Hughes, J. R. & Taylor, S. LanceOtron: a deep learning peak caller for ATAC-seq, ChIP-seq, and DNase-seq. *bioRxiv*, 2021.2001.2025.428108, doi:10.1101/2021.01.25.428108 (2021).

38 Stovner, E. B. & Saetrom, P. epic2 efficiently finds diffuse domains in ChIP-seq data. *Bioinformatics* **35**, 4392-4393, doi:10.1093/bioinformatics/btz232 (2019).

39 Quinlan, A. R. & Hall, I. M. BEDTools: a flexible suite of utilities for comparing genomic features. *Bioinformatics* **26**, 841-842, doi:10.1093/bioinformatics/btq033 (2010).

40 Ramirez, F. *et al.* deepTools2: a next generation web server for deep-sequencing data analysis. *Nucleic Acids Res* **44**, W160-165, doi:10.1093/nar/gkw257 (2016).

41 Guo, Y., Mahony, S. & Gifford, D. K. High resolution genome wide binding event finding and motif discovery reveals transcription factor spatial binding constraints. *PLoS Comput Biol* **8**, e1002638, doi:10.1371/journal.pcbi.1002638 (2012).

42 Dreos, R., Ambrosini, G., Perier, R. C. & Bucher, P. The Eukaryotic Promoter Database: expansion of EPDnew and new promoter analysis tools. *Nucleic Acids Res* **43**, D92-96, doi:10.1093/nar/gku1111 (2015).

43 Thorvaldsdottir, H., Robinson, J. T. & Mesirov, J. P. Integrative Genomics Viewer (IGV): high-performance genomics data visualization and exploration. *Brief Bioinform* **14**, 178-192, doi:10.1093/bib/bbs017 (2013).

44 Hentges, L. D. *et al.* LanceOtron: a deep learning peak caller for genome sequencing experiments. *Bioinformatics* **38**, 4255-4263, doi:10.1093/bioinformatics/btac525 (2022).

45 Love, M. I., Huber, W. & Anders, S. Moderated estimation of fold change and dispersion for RNA-seq data with DESeq2. *Genome Biol* **15**, 550, doi:10.1186/s13059-014-0550-8 (2014).

46 Bailey, T. L. STREME: accurate and versatile sequence motif discovery. *Bioinformatics* **37**, 2834-2840, doi:10.1093/bioinformatics/btab203 (2021).

47 Grant, C. E., Bailey, T. L. & Noble, W. S. FIMO: scanning for occurrences of a given motif. *Bioinformatics* **27**, 1017-1018, doi:10.1093/bioinformatics/btr064 (2011).

48 Hon, C. C. *et al.* An atlas of human long non-coding RNAs with accurate 5' ends. *Nature* **543**, 199-204, doi:10.1038/nature21374 (2017).

49 Yu, N. Y. *et al.* Complementing tissue characterization by integrating transcriptome profiling from the Human Protein Atlas and from the FANTOM5 consortium. *Nucleic Acids Res* **43**, 6787-6798, doi:10.1093/nar/gkv608 (2015).

50 Dempster, J. M. *et al.* Extracting Biological Insights from the Project Achilles Genome-Scale CRISPR Screens in Cancer Cell Lines. *bioRxiv*, 720243, doi:10.1101/720243 (2019).

51 Martin, F. J. *et al.* Ensembl 2023. *Nucleic Acids Res* **51**, D933-D941, doi:10.1093/nar/gkac958 (2023).
