# Supplementary material for: Disentangling the architectural and non-architectural functions of CTCF and cohesin in gene regulation
Source: Nat Genet. 2025 Nov 18;57(12):3137–51. doi: 10.1038/s41588-025-02404-x (PMC12695661; doi:10.1038/s41588-025-02404-x)
Supplement: Supplementary file 2 — Reporting Summary [file 41588_2025_2404_MOESM2_ESM.pdf]

nature portfolio

Corresponding author(s): Chunaram Choudhary

Last updated by author(s): Sep 18, 2025

Reporting Summary

Nature Portfolio wishes to improve the reproducibility of the work that we publish. This form provides structure for consistency and transparency in reporting. For further information on Nature Portfolio policies, see our Editorial Policies and the Editorial Policy Checklist.

Statistics

For all statistical analyses, confirm that the following items are present in the figure legend, table legend, main text, or Methods section.

n/a	Confirmed	
☐	☒	The exact sample size (*n*) for each experimental group/condition, given as a discrete number and unit of measurement
☐	☒	A statement on whether measurements were taken from distinct samples or whether the same sample was measured repeatedly
☐	☒	The statistical test(s) used AND whether they are one- or two-sided<br>*Only common tests should be described solely by name; describe more complex techniques in the Methods section.*
☐	☒	A description of all covariates tested
☐	☒	A description of any assumptions or corrections, such as tests of normality and adjustment for multiple comparisons
☐	☒	A full description of the statistical parameters including central tendency (e.g. means) or other basic estimates (e.g. regression coefficient) AND variation (e.g. standard deviation) or associated estimates of uncertainty (e.g. confidence intervals)
☐	☒	For null hypothesis testing, the test statistic (e.g. *F*, *t*, *r*) with confidence intervals, effect sizes, degrees of freedom and *P* value noted<br>*Give P values as exact values whenever suitable.*
☒	☐	For Bayesian analysis, information on the choice of priors and Markov chain Monte Carlo settings
☒	☐	For hierarchical and complex designs, identification of the appropriate level for tests and full reporting of outcomes
☐	☒	Estimates of effect sizes (e.g. Cohen's *d*, Pearson's *r*), indicating how they were calculated

Our web collection on statistics for biologists contains articles on many of the points above.

Software and code

Policy information about availability of computer code

Data collection	No software is used for data collection.
Data analysis	Cutadapt, fastqc(0.12.1), STAR(2.6.1a), Bedtools(2.23), HTseq(0.11.1), bwa meme (version 1.0.4), samtools(1.4), Lanceotron(20210215), R(4.1.1), ggplot2(3.3.5), DESeq(1.32.0), bigWigMerge, deeptools(3.5.2), IGV(2.16), Picard-tools(2.9.1), epic2(0.0.47), GEM(3.4), STREME(5.5.4), FIMO (5.5.4), higlass-python(v1.2.0)

For manuscripts utilizing custom algorithms or software that are central to the research but not yet described in published literature, software must be made available to editors and reviewers. We strongly encourage code deposition in a community repository (e.g. GitHub). See the Nature Portfolio guidelines for submitting code & software for further information.

Data

Policy information about availability of data

All manuscripts must include a data availability statement. This statement should provide the following information, where applicable:

- Accession codes, unique identifiers, or web links for publicly available datasets
- A description of any restrictions on data availability
- For clinical datasets or third party data, please ensure that the statement adheres to our policy

Raw EU-seq, ChIP-seq and ATAC-seq data generated in this study have been deposited in the NCBI Gene Expression Omnibus (GEO) under the SuperSeries accession GSE262521 (https://www.ncbi.nlm.nih.gov/geo/query/acc.cgi?acc=GSE262521). Processed read counts, peak region sets, and genome-browser tracks are provided

as supplementary files under the corresponding SubSeries accessions: ATAC-seq (GSE262516), ChIP-seq (GSE262519), and EU-seq (GSE262520). The following publicly available data were used: Mouse and Human gene annotations were from GENCODE (https://www.gencodegenes.org); FANTOM5 CAGE dataset was from ArrayExpress (https://www.ebi.ac.uk/arrayexpress/); Promoter Elements were referenced from EPD (https://epd.epfl.ch//index.php); Motif references were downloaded from JASPAR (https://jaspar.genereg.net/). DepMap datasets were downloaded from the DepMap portal (https://depmap.org). The orthologue reference was downloaded from (https://www.ensembl.org/info/data/biomart).
The following publicly available sequencing datasets were reanalyzed: RNA-seq in CTCFAID ESC with or without depletion of CTCFAID, and with or without, overexpressing BORIS or CTCF-BORIS chimera (GSE140363). EU-seq in ESC treated without and with A-485 (GSE146328). ChIP-exo-seq in ESC on CTCF (GSE98671). ChIP-seq in NPC on CTCF and Rad21 (GSE262551). ChIP-seq in ESC on H3K4me3 (GSE146328), TBP (GSE146328), H3K27me3 (GSE186349), Input control (GSE146328), CTCF (GSE178982), Rad21 (GSE178982). Following CTCF ChIP-seq and ChIP-exo-seq data in a variety of tissues and cell lines were reanalyzed. Mouse: 3134 cells (GSE61236), 3T3-L1 cells (GSE95533), AML12 cells(GSE95116), splenic B cells (GSE44637), bone marrow derived macrophages (GSE189975), bone marrow (GSE49847), brain (GSE114606), brown preadipocytes (GSE74189), cerebellum (GSE49847), CFUMk cells(GSE156074), CH12 cells (GSE49847), CMP cells (GSE159503), cortex (GSE49847), distal forelimbs (GSE101714), DP cells (GSE141223), ECOMG-derived neutrophils (GSE93127), embryoid bodies (GSE119874), EpiLC cells (GSE183828), erythroid cells (GSE142006), erythroid progenitor cells (GSE150415), female germline stem cells (GSE137771), forebrain (GSE127870), G1E cells (GSE156074), GMP cells (GSE156074), GSC cells (GSE183828), GSCLC cells (GSE183828), heart (GSE91813), Hepa-1c1c7 cells (GSE154387), HPC7 cells (GSE48086), HSPC cells (GSE131583), kidney (GSE49847), liver (GSE91731), MEF cells (GSE99197), MEL cells (GSE181234), mature olfactory sensory neurons (GSE112153), nephron progenitor cells (GSE90016), anterior neural progenitor cells (GSE160654), pancreas (GSE59119), Patski cells (GSE59779), pro-B cells (GSE109909), round spermatids (GSE70764), small intestine (GSE49847), spinal motor neurons (GSE196170), spleen (GSE111772), splenic plasmablasts (GSE44637), stomach (GSE91488), testis (GSE49847), thymus (GSE180937), trophoblast stem cells (GSE110950). Human: A-673 cells (GSE185132), adrenal gland (GSE106071), ascending.aorta (GSE143103), astrocytes (GSE30263), B cell (GSE206145), bipolar neuron (GSE96269), 5637 cells (GSE193886), brain (GSE209256), brain microvascular endothelial cell (GSE30263), breast epithelium (GSE105608), CLB-Ga cells (GSE224242), CMP cells (GSE231486), colonic mucosa (GSE209074), coronary artery (GSE127477), DLD-1 cells (GSE142746), esophagus muscularis mucosa (GSE142970) , esophagus squamous epithelium (GSE105931), gastrocnemius medialis (GSE143020), gastroesophageal sphincter (GSE127521), GMP cells (GSE231486), Gp5d cells (GSE180150), ESC (GSE211101), ESC-derived SC-beta cells (GSE211101), HAP1 cells (GSE180691), HCT116 cells (GSE184106), heart right ventricle (GSE175279), HEK293T cells (GSE206145), HeLa S3 cells (GSE32883), HepG2 cells (GSE30226), HT1080 cells (GSE153869), Jurkat cells (GSE130140), K562 cells (GSE180175), Kidney (GSE33213), Kuramochi cells (GSE152885), L1207 cells (GSE193886), left lung (GSE188029), left ventricle myocardium inferior (GSE187266), liver (GSE127549), lower leg skin (GSE105391), limbal stem cells (GSE192625), lung (GSE33213), MCF 10A cell (GSE183381), MCF7 cell (GSE181460), MEP cells (GSE231486), mucosa descending colon (GSE208481), Mutu-1 cells (GSE160973), pancreas (GSE174993), Peyers patch (GSE105594), PLC/PRF/5 cells (GSE209849), 22Rv1 cells (GSE200168), psoas muscle (GSE209062), right atrium auricular (GSE127378), RPE cells (GSE196727), RT-112 cells (GSE193886), SD48 cells (GSE193886), SH-SY5Y cells (GSE141278), sigmoid colon (GSE105852), suprapubic skin (GSE139782), testis (GSE105739), thoracic aorta (GSE127422), thyroid gland (GSE105921), tibial artery (GSE105707), tibial nerve (GSE105554), U2OS cells (GSE175731), vagina (GSE105477). The following publicly available processed files were used for data visualization.
Micro-C data in RAD21AID ESC with or without IAA treatment (GSE178982) (GSE178982_RAD21-UT_pool.mcool and GSE178982_RAD21-AID_pool.mcool).

Research involving human participants, their data, or biological material

Policy information about studies with human participants or human data. See also policy information about sex, gender (identity/presentation), and sexual orientation and race, ethnicity and racism.

Reporting on sex and gender	Not relevant.
Reporting on race, ethnicity, or other socially relevant groupings	Not relevant.
Population characteristics	Not relevant.
Recruitment	Not relevant.
Ethics oversight	Not relevant.

Note that full information on the approval of the study protocol must also be provided in the manuscript.

Field-specific reporting

Please select the one below that is the best fit for your research. If you are not sure, read the appropriate sections before making your selection.

☒ Life sciences ☐ Behavioural & social sciences ☐ Ecological, evolutionary & environmental sciences

For a reference copy of the document with all sections, see nature.com/documents/nr-reporting-summary-flat.pdf

Life sciences study design

All studies must disclose on these points even when the disclosure is negative.

Sample size	No statistical methods were used to pre-determine sample sizes but our sample sizes are similar to those reported in previous publications (PMID 35410381, PMID 36471071, PMID: 34002095).
Data exclusions	No data were excluded.
Replication	All attempts to replicate the results were successful. ATAC-seq and ChIP-seq were each performed in two biological replicates. For analyses of expression changes following CTCF depletion, both stranded and non-stranded EU-seq were conducted with two biological replicates.

Expression changes after RAD21 depletion or CTCF YF-AA mutant were also assessed in two biological replicates. To evaluate the robustness of EU-seq measurements for RAD21 depletion, we analyzed an additional 12 biological replicates. Transcriptome changes after bTAG-ΔN CTCF and/or dTAG CTCF-ΔC depletion were assessed using two biological replicates. Protein depletion of RAD21 and CTCF was confirmed by western blotting and/or quantitative microscopy with the following designs: RAD21 depletion—one biological replicate at two time points; CTCF depletion—one biological replicate at two time points; CTCF YF-AA mutant—one biological replicate at one time point; bTAG-ΔN CTCF and/or dTAG CTCF-ΔC—three biological replicates at one time point.

Randomization	The study did not involve animal or human participants. Random allocation did not apply because samples were not subjected to co- or multivariate analysis.
Blinding	Control and treatment cells were cultured in parallel under identical conditions, and all procedures other than the intended treatment were performed identically. Library preparation and sequencing followed the same protocols for all samples as described in the method section.

Reporting for specific materials, systems and methods

We require information from authors about some types of materials, experimental systems and methods used in many studies. Here, indicate whether each material, system or method listed is relevant to your study. If you are not sure if a list item applies to your research, read the appropriate section before selecting a response.

Materials & experimental systems

n/a	Involved in the study	
☐	☒	Antibodies
☐	☒	Eukaryotic cell lines
☒	☐	Palaeontology and archaeology
☒	☐	Animals and other organisms
☒	☐	Clinical data
☒	☐	Dual use research of concern
☒	☐	Plants

Methods

n/a	Involved in the study	
☐	☒	ChIP-seq
☒	☐	Flow cytometry
☒	☐	MRI-based neuroimaging

Antibodies

Antibodies used

ChIP-seq
Rpb1 NTD (D8L4Y) Rabbit mAb #14958, Cell Signaling Technology, 5ug

Western blotting
CTCF (D31H2) XP Rabbit mAb #3418, Cell Signaling Technology, 1:1000
Histone H3 (D1H2) XP Rabbit mAb #4499, Cell Signaling Technology
Anti-GAPDH Antibody #ABS16, Sigma-Aldrich

Immunostaining
CTCF (D31H2) XP Rabbit mAb #3418, Cell Signaling Technology, 1:1000
ALFA Recombinant anti-ALFA Antibody #N1581, NanoTag Biotechnologies, 1:500
Goat anti-Mouse IgG (H+L) Cross-Adsorbed Secondary Antibody, Alexa Flur 647, A-21235, Invitrogen

Validation

The following quality assurance were provided on the manufacture's websites.
Anti-Rpb1 NTD ab was tested by ChIP-seq in HeLa cells. According to the provider, Rpb1 NTD (D8L4Y) Rabbit mAb recognizes endogenous levels of total Rpb1 protein at the amino terminal domain (NTD). (https://www.cellsignal.com/products/primary-antibodies/rpb1-ntd-d8l4y-rabbit-mab/14958)

Anti-CTCF ab was tested by western blot in HeLa, NIH3T3, C6, and COS cells, and also by immunofluorescent analysis of HCT-116 cells. Acoording to the provider, CTCF (D31H2) XP® Rabbit mAb detects endogenous levels of total CTCF protein. This antibody does not cross-react with BORIS. This antibody nonspecifically labels the lamina propria of small intestine in fixed frozen mouse tissue by immunofluorescence. (https://www.cellsignal.com/products/primary-antibodies/ctcf-d31h2-xp-rabbit-mab/3418)

Anti-H3 ab was tested by western blot in HeLa, NIH3T3, C6, and COS cells. According to the provider, Histone H3 (D1H2) XP® Rabbit mAb detects endogenous levels of total Histone H3 protein, including isoforms H3.1, H3.2, and H3.3. This antibody also detects the Histone H3 variant CENP-A. This antibody does not cross-react with other core histones. (https://www.cellsignal.com/products/primary-antibodies/histone-h3-d1h2-xp-rabbit-mab/4499)

Anti-GAPDH antibody was evaluated by Western blot in HEK293 cell lysates. According to the provider, This antibody is supported by peer reviewed publications and reliably detects Glyceraldehyde-3-Phosphate Dehydrogenase (GAPDH) is validated for use in WB. (https://www.sigmaaldrich.com/DK/en/product/mm/abs16)

Eukaryotic cell lines

Policy information about cell lines and Sex and Gender in Research

Cell line source(s)

Mouse embryonic stem cells (mESC; E14TG2a; Sigma-Aldrich, Cat# 08021401) were purchased from Sigma-Aldrich. RAD21-

Cell line source(s)	AID mESCs were provided by Dr Rob Klose. Ctcf-mAID-GFP mESCs were provided by Drs Elphège P. Nora and Benoit G. Bruneau.
Authentication	The identity of ESC and NSC were authenticated by the expression of cell-type-specific markers by EU-seq.
Mycoplasma contamination	Cell lines were tested for mycoplasma contamination every 2-3 months, and were confirmed mycoplasma negative.
Commonly misidentified lines (See ICLAC register)	None of the used cell lines are listed in the commonly misidentified lines.

Plants

Seed stocks	Not relevant.
Novel plant genotypes	Not relevant.
Authentication	Not relevant.

ChIP-seq

Data deposition

☒ Confirm that both raw and final processed data have been deposited in a public database such as GEO.

☒ Confirm that you have deposited or provided access to graph files (e.g. BED files) for the called peaks.

Data access links
May remain private before publication.

https://www.ncbi.nlm.nih.gov/geo/query/acc.cgi?acc=GSE262521

Files in database submission

ATAC-seq
ATACSeq.dTAG.CTCF.ESC_Ctrl_TC0_CC30_1_R1.fastq.gz
ATACSeq.dTAG.CTCF.ESC_Ctrl_TC0_CC30_1_R2.fastq.gz
ATACSeq.dTAG.CTCF.ESC_Ctrl_TC0_CC30_2_R1.fastq.gz
ATACSeq.dTAG.CTCF.ESC_Ctrl_TC0_CC30_2_R2.fastq.gz
ATACSeq.dTAG.CTCF.ESC_dTAG13_100nM_TC180_CC30_1_R1.fastq.gz
ATACSeq.dTAG.CTCF.ESC_dTAG13_100nM_TC180_CC30_1_R2.fastq.gz
ATACSeq.dTAG.CTCF.ESC_dTAG13_100nM_TC180_CC30_2_R1.fastq.gz
ATACSeq.dTAG.CTCF.ESC_dTAG13_100nM_TC180_CC30_2_R2.fastq.gz

ChIP-seq
GFP.dTAG.CTCF.ESC_Ctrl_TC0_Pol2.D8L4Y_CC23_1.fastq.gz
GFP.dTAG.CTCF.ESC_Ctrl_TC0_Pol2.D8L4Y_CC25_1.fastq.gz
GFP.dTAG.CTCF.ESC_dtag13_100nM_TC240_Pol2.D8L4Y_CC23_1.fastq.gz
GFP.dTAG.CTCF.ESC_dtag13_100nM_TC240_Pol2.D8L4Y_CC25_1.fastq.gz

EU-seq
EUSeq.GFP.dTAG.CTCF.Y226A.F228A.ESC_Ctrl_TC0_spike.dm_CC37_1.fastq.gz
EUSeq.GFP.dTAG.CTCF.Y226A.F228A.ESC_Ctrl_TC0_spike.dm_CC37_2.fastq.gz
EUSeq.GFP.dTAG.CTCF.Y226A.F228A.ESC_dTAG13_200nM_TC120_spike.dm_CC37_1.fastq.gz
EUSeq.GFP.dTAG.CTCF.Y226A.F228A.ESC_dTAG13_200nM_TC120_spike.dm_CC37_2.fastq.gz
EUSeq.GFP.dTAG.CTCF.Y226A.F228A.ESC_dTAG13_200nM_TC240_spike.dm_CC37_1.fastq.gz
EUSeq.GFP.dTAG.CTCF.Y226A.F228A.ESC_dTAG13_200nM_TC240_spike.dm_CC37_2.fastq.gz

EUSeq.GFP.AID.CTCF.ESC_Ctrl_TC0_EU.CC5_1.fastq.gz
EUSeq.GFP.AID.CTCF.ESC_Ctrl_TC0_EU.CC5_2.fastq.gz
EUSeq.GFP.AID.CTCF.ESC_Ctrl_TC0_strand_CC26_1.fastq.gz
EUSeq.GFP.AID.CTCF.ESC_Ctrl_TC0_strand_CC29_1.fastq.gz
EUSeq.GFP.AID.CTCF.ESC_IAA_500uM_TC360_EU.CC5_1.fastq.gz
EUSeq.GFP.AID.CTCF.ESC_IAA_500uM_TC360_EU.CC5_2.fastq.gz
EUSeq.GFP.AID.CTCF.ESC_IAA_500uM_TC360_strand_CC26_1.fastq.gz
EUSeq.GFP.AID.CTCF.ESC_IAA_500uM_TC360_strand_CC29_1.fastq.gz

EUSeq.GFP.AID.Rad21.ESC_Ctrl_TC0_CC24_1.fastq.gz
EUSeq.GFP.AID.Rad21.ESC_Ctrl_TC0_CC24_2.fastq.gz
EUSeq.GFP.AID.Rad21.ESC_IAA_500uM_TC240_CC24_1.fastq.gz
EUSeq.GFP.AID.Rad21.ESC_IAA_500uM_TC240_CC24_2.fastq.gz

EUSeq.GFP.AID.Rad21.NSC_Ctrl_TC0_CC56_1.fastq.gz
EUSeq.GFP.AID.Rad21.NSC_Ctrl_TC0_CC56_2.fastq.gz
EUSeq.GFP.AID.Rad21.NSC_IAA_500uM_TC60_CC56_1.fastq.gz
EUSeq.GFP.AID.Rad21.NSC_IAA_500uM_TC60_CC56_2.fastq.gz
EUSeq.GFP.AID.Rad21.NSC_IAA_500uM_TC120_CC56_1.fastq.gz
EUSeq.GFP.AID.Rad21.NSC_IAA_500uM_TC120_CC56_2.fastq.gz
EUSeq.GFP.AID.Rad21.NSC_IAA_500uM_TC240_CC56_1.fastq.gz
EUSeq.GFP.AID.Rad21.NSC_IAA_500uM_TC240_CC56_2.fastq.gz
EUSeq.GFP.AID.Rad21.NSC_A485_10uM_TC60_CC56_1.fastq.gz
EUSeq.GFP.AID.Rad21.NSC_A485_10uM_TC60_CC56_2.fastq.gz

EUSeq.GFP.AID.Rad21.ESC_Ctrl_TC0_CC52_1.fastq.gz
EUSeq.GFP.AID.Rad21.ESC_Ctrl_TC0_CC53_1.fastq.gz
EUSeq.GFP.AID.Rad21.ESC_Ctrl_TC0_CC53_2.fastq.gz
EUSeq.GFP.AID.Rad21.ESC_Ctrl_TC0_CC53_3.fastq.gz
EUSeq.GFP.AID.Rad21.ESC_Ctrl_TC0_CC53_4.fastq.gz
EUSeq.GFP.AID.Rad21.ESC_Ctrl_TC0_CC53_5.fastq.gz
EUSeq.GFP.AID.Rad21.ESC_Ctrl_TC0_CC53_6.fastq.gz
EUSeq.GFP.AID.Rad21.ESC_Ctrl_TC0_CC53_7.fastq.gz
EUSeq.GFP.AID.Rad21.ESC_Ctrl_TC0_CC53_8.fastq.gz
EUSeq.GFP.AID.Rad21.ESC_Ctrl_TC0_CC53_9.fastq.gz
EUSeq.GFP.AID.Rad21.ESC_Ctrl_TC0_CC53_10.fastq.gz
EUSeq.GFP.AID.Rad21.ESC_Ctrl_TC0_CC53_11.fastq.gz

EUSeq.GFP.AID.Rad21.ESC_IAA_500uM_TC240_CC52_1.fastq.gz
EUSeq.GFP.AID.Rad21.ESC_IAA_500uM_TC240_CC53_1.fastq.gz
EUSeq.GFP.AID.Rad21.ESC_IAA_500uM_TC240_CC53_2.fastq.gz
EUSeq.GFP.AID.Rad21.ESC_IAA_500uM_TC240_CC53_3.fastq.gz
EUSeq.GFP.AID.Rad21.ESC_IAA_500uM_TC240_CC53_4.fastq.gz
EUSeq.GFP.AID.Rad21.ESC_IAA_500uM_TC240_CC53_5.fastq.gz
EUSeq.GFP.AID.Rad21.ESC_IAA_500uM_TC240_CC53_6.fastq.gz
EUSeq.GFP.AID.Rad21.ESC_IAA_500uM_TC240_CC53_7.fastq.gz
EUSeq.GFP.AID.Rad21.ESC_IAA_500uM_TC240_CC53_8.fastq.gz
EUSeq.GFP.AID.Rad21.ESC_IAA_500uM_TC240_CC53_9.fastq.gz
EUSeq.GFP.AID.Rad21.ESC_IAA_500uM_TC240_CC53_10.fastq.gz
EUSeq.GFP.AID.Rad21.ESC_IAA_500uM_TC240_CC53_11.fastq.gz

EUSeq.GFP.dTAG.1.577aa.CTCF_bTAG.Alfa.266.736aa.CTCF.ESC_Ctrl_TC0_strand_CC71_1.fastq.gz
EUSeq.GFP.dTAG.1.577aa.CTCF_bTAG.Alfa.266.736aa.CTCF.ESC_Ctrl_TC0_strand_CC71_2.fastq.gz
EUSeq.GFP.dTAG.1.577aa.CTCF_bTAG.Alfa.266.736aa.CTCF.ESC_AGB1_100nM_TC120_strand_CC71_1.fastq.gz
EUSeq.GFP.dTAG.1.577aa.CTCF_bTAG.Alfa.266.736aa.CTCF.ESC_AGB1_100nM_TC120_strand_CC71_2.fastq.gz
EUSeq.GFP.dTAG.1.577aa.CTCF_bTAG.Alfa.266.736aa.CTCF.ESC_dTAG13_200nM_TC120_strand_CC71_1.fastq.gz
EUSeq.GFP.dTAG.1.577aa.CTCF_bTAG.Alfa.266.736aa.CTCF.ESC_dTAG13_200nM_TC120_strand_CC71_2.fastq.gz
EUSeq.GFP.dTAG.1.577aa.CTCF_bTAG.Alfa.266.736aa.CTCF.ESC_dTAG13_200nM_AGB1_100nM_TC120_strand_CC71_1.fastq.gz
EUSeq.GFP.dTAG.1.577aa.CTCF_bTAG.Alfa.266.736aa.CTCF.ESC_dTAG13_200nM_AGB1_100nM_TC120_strand_CC71_2.fastq.gz

Genome browser session (e.g. UCSC)

NA

Methodology

Replicates

Two biological replicates were performed for both ATAC-seq and ChIP-seq experiments. For the analysis of expression changes following CTCF depletion, two biological replicates were conducted in both stranded EU-seq and non-stranded EU-seq experiments. For the analysis of expression changes after RAD21 depletion or the introduction of the YF-AA CTCF mutant, two biological replicates were performed. To assess the robustness of EU-seq experiments, expression changes after RAD21 depletion analyzed using 12 additional biological replicates. Transcriptome changes after bTAG_ΔNCtcf and/or dTAGCtcfΔC depletion were performed using two biological replicates.

Sequencing depth

ATAC-seq
ATACSeq.dTAG.CTCF.ESC_Ctrl_TC0_CC30_1_R1.fastq.gz, ATACSeq.dTAG.CTCF.ESC_Ctrl_TC0_CC30_1_R2.fastq.gz, total:95504203, unique:48863451, 41bp, paired-end
ATACSeq.dTAG.CTCF.ESC_Ctrl_TC0_CC30_2_R1.fastq.gz, ATACSeq.dTAG.CTCF.ESC_Ctrl_TC0_CC30_2_R2.fastq.gz, total:91042591, unique:45519571, 41bp, paired-end
ATACSeq.dTAG.CTCF.ESC_dTAG13_100nM_TC180_CC30_1_R1.fastq.gz,
ATACSeq.dTAG.CTCF.ESC_dTAG13_100nM_TC180_CC30_1_R2.fastq.gz, total:91098130, unique:44343561, 41bp, paired-end
ATACSeq.dTAG.CTCF.ESC_dTAG13_100nM_TC180_CC30_2_R1.fastq.gz,
ATACSeq.dTAG.CTCF.ESC_dTAG13_100nM_TC180_CC30_2_R2.fastq.gz, total:85262149, unique:43015138, 41bp, paired-end

ChIP-seq
GFP.dTAG.CTCF.ESC_Ctrl_TC0_Pol2.D8L4Y_CC23_1.fastq.gz, total:37738410, unique:26519883, 101bp, single-end
GFP.dTAG.CTCF.ESC_Ctrl_TC0_Pol2.D8L4Y_CC25_1.fastq.gz, total:34739334, unique:25402293, 101bp, single-end

GFP.dTAG.CTCF.ESC_dtag13_100nM_TC240_Pol2.D8L4Y_CC23_1.fastq.gz, total:37554806, unique:26866053, 101bp, single-end
GFP.dTAG.CTCF.ESC_dtag13_100nM_TC240_Pol2.D8L4Y_CC25_1.fastq.gz, total:33599053, unique:24715268, 101bp, single-end

EU-seq
EUSeq.GFP.dTAG.CTCF.Y226A.F228A.ESC_Ctrl_TC0_spike.dm_CC37_1.fastq.gz, total:14419123, mapped to the mouse genome (excluding rRNA, tRNA):6738367, 82bp, single-end
EUSeq.GFP.dTAG.CTCF.Y226A.F228A.ESC_Ctrl_TC0_spike.dm_CC37_2.fastq.gz, total:38526836, mapped to the mouse genome (excluding rRNA, tRNA):17389892, 82bp, single-end
EUSeq.GFP.dTAG.CTCF.Y226A.F228A.ESC_dTAG13_200nM_TC120_spike.dm_CC37_1.fastq.gz, total:15651533, mapped to the mouse genome (excluding rRNA, tRNA):7158643, 82bp, single-end
EUSeq.GFP.dTAG.CTCF.Y226A.F228A.ESC_dTAG13_200nM_TC120_spike.dm_CC37_2.fastq.gz, total:34666193, mapped to the mouse genome (excluding rRNA, tRNA):15634422, 82bp, single-end
EUSeq.GFP.dTAG.CTCF.Y226A.F228A.ESC_dTAG13_200nM_TC240_spike.dm_CC37_1.fastq.gz, total:26202524, mapped to the mouse genome (excluding rRNA, tRNA):12041967, 82bp, single-end
EUSeq.GFP.dTAG.CTCF.Y226A.F228A.ESC_dTAG13_200nM_TC240_spike.dm_CC37_2.fastq.gz, total:36482931, mapped to the mouse genome (excluding rRNA, tRNA):16347731, 82bp, single-end

EUSeq.GFP.AID.CTCF.ESC_Ctrl_TC0_EU.CC5_1.fastq.gz, total:30248062, mapped (excluding rRNA, tRNA):23027586, 75bp, single-end
EUSeq.GFP.AID.CTCF.ESC_Ctrl_TC0_EU.CC5_2.fastq.gz, total:31174220, mapped (excluding rRNA, tRNA):24086800, 75bp, single-end
EUSeq.GFP.AID.CTCF.ESC_Ctrl_TC0_strand_CC26_1.fastq.gz, total:35113121, mapped (excluding rRNA, tRNA):18546823, 101bp, single-end
EUSeq.GFP.AID.CTCF.ESC_Ctrl_TC0_strand_CC29_1.fastq.gz, total:62355181, mapped (excluding rRNA, tRNA):35663422, 101bp, single-end
EUSeq.GFP.AID.CTCF.ESC_IAA_500uM_TC360_EU.CC5_1.fastq.gz, total:32874432, mapped (excluding rRNA, tRNA):24797490, 75bp, single-end
EUSeq.GFP.AID.CTCF.ESC_IAA_500uM_TC360_EU.CC5_2.fastq.gz, total:30707213, mapped (excluding rRNA, tRNA):21445919, 75bp, single-end
EUSeq.GFP.AID.CTCF.ESC_IAA_500uM_TC360_strand_CC26_1.fastq.gz, total:31187672, mapped (excluding rRNA, tRNA):15302948, 101bp, single-end
EUSeq.GFP.AID.CTCF.ESC_IAA_500uM_TC360_strand_CC29_1.fastq.gz, total:57686132, mapped (excluding rRNA, tRNA):31351029, 101bp, single-end

EUSeq.GFP.AID.Rad21.ESC_Ctrl_TC0_CC24_1.fastq.gz, total:27205836, mapped (excluding rRNA, tRNA):15222440, 101bp, single-end
EUSeq.GFP.AID.Rad21.ESC_Ctrl_TC0_CC24_2.fastq.gz, total:29298620, mapped (excluding rRNA, tRNA):17367617, 101bp, single-end
EUSeq.GFP.AID.Rad21.ESC_IAA_500uM_TC240_CC24_1.fastq.gz, total:28119926, mapped (excluding rRNA, tRNA):15702675, 101bp, single-end
EUSeq.GFP.AID.Rad21.ESC_IAA_500uM_TC240_CC24_2.fastq.gz, total:32429251, mapped (excluding rRNA, tRNA):17425766, 101bp, single-end

EUSeq.GFP.AID.Rad21.NSC_Ctrl_TC0_CC56_1.fastq.gz, total: 48987777, mapped (excluding rRNA, tRNA):25478206, 132bp, single-end
EUSeq.GFP.AID.Rad21.NSC_Ctrl_TC0_CC56_2.fastq.gz, total: 54143947, mapped (excluding rRNA, tRNA):28565765, 132bp, single-end
EUSeq.GFP.AID.Rad21.NSC_IAA_500uM_TC60_CC56_1.fastq.gz, total: 50431987, mapped (excluding rRNA, tRNA):23210749, 132bp, single-end
EUSeq.GFP.AID.Rad21.NSC_IAA_500uM_TC60_CC56_2.fastq.gz, total: 50035877, mapped (excluding rRNA, tRNA):26043886, 132bp, single-end
EUSeq.GFP.AID.Rad21.NSC_IAA_500uM_TC120_CC56_1.fastq.gz, total: 57933246, mapped (excluding rRNA, tRNA):26530518, 132bp, single-end
EUSeq.GFP.AID.Rad21.NSC_IAA_500uM_TC120_CC56_2.fastq.gz, total: 47171992, mapped (excluding rRNA, tRNA):14714775, 132bp, single-end
EUSeq.GFP.AID.Rad21.NSC_IAA_500uM_TC240_CC56_1.fastq.gz, total: 53498665, mapped (excluding rRNA, tRNA):26173221, 132bp, single-end
EUSeq.GFP.AID.Rad21.NSC_IAA_500uM_TC240_CC56_2.fastq.gz, total: 50727296, mapped (excluding rRNA, tRNA):23934216, 132bp, single-end
EUSeq.GFP.AID.Rad21.NSC_A485_10uM_TC60_CC56_1.fastq.gz, total: 48556419, mapped (excluding rRNA, tRNA):23455010, 132bp, single-end
EUSeq.GFP.AID.Rad21.NSC_A485_10uM_TC60_CC56_2.fastq.gz, total: 57198713, mapped (excluding rRNA, tRNA):24723013, 132bp, single-end

EUSeq.GFP.AID.Rad21.ESC_Ctrl_TC0_CC52_1.fastq.gz, total: 28219652, mapped (excluding rRNA, tRNA):13541043, 132bp, single-end
EUSeq.GFP.AID.Rad21.ESC_Ctrl_TC0_CC53_1.fastq.gz, total: 58460033, mapped (excluding rRNA, tRNA):27382270, 82bp, single-end
EUSeq.GFP.AID.Rad21.ESC_Ctrl_TC0_CC53_2.fastq.gz, total: 53798231, mapped (excluding rRNA, tRNA):24170540, 82bp, single-end
EUSeq.GFP.AID.Rad21.ESC_Ctrl_TC0_CC53_3.fastq.gz, total: 61775977, mapped (excluding rRNA, tRNA):30367238, 82bp, single-end
EUSeq.GFP.AID.Rad21.ESC_Ctrl_TC0_CC53_4.fastq.gz, total: 56607381, mapped (excluding rRNA, tRNA):26599994, 82bp, single-end
EUSeq.GFP.AID.Rad21.ESC_Ctrl_TC0_CC53_5.fastq.gz, total: 71895791, mapped (excluding rRNA, tRNA):33218201, 82bp, single-end
EUSeq.GFP.AID.Rad21.ESC_Ctrl_TC0_CC53_6.fastq.gz, total: 59612965, mapped (excluding rRNA, tRNA):27150564, 82bp, single-end
EUSeq.GFP.AID.Rad21.ESC_Ctrl_TC0_CC53_7.fastq.gz, total: 70230572, mapped (excluding rRNA, tRNA):31681225, 82bp, single-end
EUSeq.GFP.AID.Rad21.ESC_Ctrl_TC0_CC53_8.fastq.gz, total: 54155638, mapped (excluding rRNA, tRNA):25266713, 82bp, single-end
EUSeq.GFP.AID.Rad21.ESC_Ctrl_TC0_CC53_9.fastq.gz, total: 54791813, mapped (excluding rRNA, tRNA):25840812, 82bp, single-end
EUSeq.GFP.AID.Rad21.ESC_Ctrl_TC0_CC53_10.fastq.gz, total: 57915265, mapped (excluding rRNA, tRNA):27646378, 82bp, single-end
EUSeq.GFP.AID.Rad21.ESC_Ctrl_TC0_CC53_11.fastq.gz, total: 58831257, mapped (excluding rRNA, tRNA):27144694, 82bp, single-end

EUSeq.GFP.AID.Rad21.ESC_IAA_500uM_TC240_CC52_1.fastq.gz, total: 30286974, mapped (excluding rRNA, tRNA):14545398, 132bp, single-end

EUSeq.GFP.AID.Rad21.ESC_IAA_500uM_TC240_CC53_1.fastq.gz, total: 57175734, mapped (excluding rRNA, tRNA):27015488, 82bp, single-end
EUSeq.GFP.AID.Rad21.ESC_IAA_500uM_TC240_CC53_2.fastq.gz, total: 56839342, mapped (excluding rRNA, tRNA):26485244, 82bp, single-end
EUSeq.GFP.AID.Rad21.ESC_IAA_500uM_TC240_CC53_3.fastq.gz, total: 55248304, mapped (excluding rRNA, tRNA):25509358, 82bp, single-end
EUSeq.GFP.AID.Rad21.ESC_IAA_500uM_TC240_CC53_4.fastq.gz, total: 57091660, mapped (excluding rRNA, tRNA):26336268, 82bp, single-end
EUSeq.GFP.AID.Rad21.ESC_IAA_500uM_TC240_CC53_5.fastq.gz, total: 80134100, mapped (excluding rRNA, tRNA):37153615, 82bp, single-end
EUSeq.GFP.AID.Rad21.ESC_IAA_500uM_TC240_CC53_6.fastq.gz, total: 61141076, mapped (excluding rRNA, tRNA):28136519, 82bp, single-end
EUSeq.GFP.AID.Rad21.ESC_IAA_500uM_TC240_CC53_7.fastq.gz, total: 53578200, mapped (excluding rRNA, tRNA):23732150, 82bp, single-end
EUSeq.GFP.AID.Rad21.ESC_IAA_500uM_TC240_CC53_8.fastq.gz, total: 61845713, mapped (excluding rRNA, tRNA):28202924, 82bp, single-end
EUSeq.GFP.AID.Rad21.ESC_IAA_500uM_TC240_CC53_9.fastq.gz, total: 59599989, mapped (excluding rRNA, tRNA):27559979, 82bp, single-end
EUSeq.GFP.AID.Rad21.ESC_IAA_500uM_TC240_CC53_10.fastq.gz, total: 55666119, mapped (excluding rRNA, tRNA):26191806, 82bp, single-end
EUSeq.GFP.AID.Rad21.ESC_IAA_500uM_TC240_CC53_11.fastq.gz, total: 54016766, mapped (excluding rRNA, tRNA):24467447, 82bp, single-end

EUSeq.GFP.dTAG.1.577aa.CTCF_bTAG.Alfa.266.736aa.CTCF.ESC_Ctrl_TC0_strand_CC71_1.fastq.gz, total: 30853200, mapped (excluding rRNA, tRNA):12928287, 132bp, single-end
EUSeq.GFP.dTAG.1.577aa.CTCF_bTAG.Alfa.266.736aa.CTCF.ESC_Ctrl_TC0_strand_CC71_2.fastq.gz, total: 30750153, mapped (excluding rRNA, tRNA):13868505, 132bp, single-end
EUSeq.GFP.dTAG.1.577aa.CTCF_bTAG.Alfa.266.736aa.CTCF.ESC_AGB1_100nM_TC120_strand_CC71_1.fastq.gz, total: 27530797, mapped (excluding rRNA, tRNA):13226384, 132bp, single-end
EUSeq.GFP.dTAG.1.577aa.CTCF_bTAG.Alfa.266.736aa.CTCF.ESC_AGB1_100nM_TC120_strand_CC71_2.fastq.gz, total: 29982928, mapped (excluding rRNA, tRNA):15474652, 132bp, single-end
EUSeq.GFP.dTAG.1.577aa.CTCF_bTAG.Alfa.266.736aa.CTCF.ESC_dTAG13_200nM_TC120_strand_CC71_1.fastq.gz, total: 31059148, mapped (excluding rRNA, tRNA):11848765, 132bp, single-end
EUSeq.GFP.dTAG.1.577aa.CTCF_bTAG.Alfa.266.736aa.CTCF.ESC_dTAG13_200nM_TC120_strand_CC71_2.fastq.gz, total: 29079519, mapped (excluding rRNA, tRNA):15908468, 132bp, single-end
EUSeq.GFP.dTAG.1.577aa.CTCF_bTAG.Alfa.266.736aa.CTCF.ESC_dTAG13_200nM_AGB1_100nM_TC120_strand_CC71_1.fastq.gz, total: 37541169, mapped (excluding rRNA, tRNA):17875249, 132bp, single-end
EUSeq.GFP.dTAG.1.577aa.CTCF_bTAG.Alfa.266.736aa.CTCF.ESC_dTAG13_200nM_AGB1_100nM_TC120_strand_CC71_2.fastq.gz, total: 27654266, mapped (excluding rRNA, tRNA):14149272, 132bp, single-end

Antibodies

The following antibodies were used for ChIP-seq.
Rpb1 NTD (D8L4Y) Rabbit mAb #14958, Cell Signaling Technology

Peak calling parameters

Reads were mapped to the reference genome by using bwa aln with default parameters (BWA version 0.7.10). Multi-mapped reads, duplicated reads, or reads with more than three mismatches were removed by samtools. Reads mapped to the DAC Blacklisted Regions (https://www.encodeproject.org/annotations/ENCSR636HFF/) were omitted from the downstream analysis. Peak regions were called using LanceOtron with default model (wide-and-deep_jan-2021) (doi: https://doi.org/10.1101/2021.01.25.428108). The poorly enriched peaks of maximum peak height < 8 reads mapped per million (rpm) were omitted.

Data quality

FASTQC was used for quality check of sequencing reads.

Software

Cutadapt, fastqc(0.12.1), STAR(2.6.1a), Bedtools(2.23), HTseq(0.11.1), bwa meme (version 1.0.4), samtools(1.4), Lanceotron(20210215), R(4.1.1), ggplot2(3.3.5), DESeq(1.32.0), bigWigMerge, deeptools(3.5.2), IGV(2.16), Picard-tools(2.9.1), epic2(0.0.47), GEM(3.4), STREME(5.5.4), FIMO (5.5.4), higlass-python(v1.2.0)
