# Supplementary material for: Disentangling the architectural and non-architectural functions of CTCF and cohesin in gene regulation
Source: Nat Genet. 2025 Nov 18;57(12):3137–51. doi: 10.1038/s41588-025-02404-x (PMC12695661; doi:10.1038/s41588-025-02404-x)
Supplement: Supplementary file 9 — Source data of Extended Data Fig. 7a. [file 41588_2025_2404_MOESM9_ESM.pdf]

Image Source Data of Extended Data Fig.7a

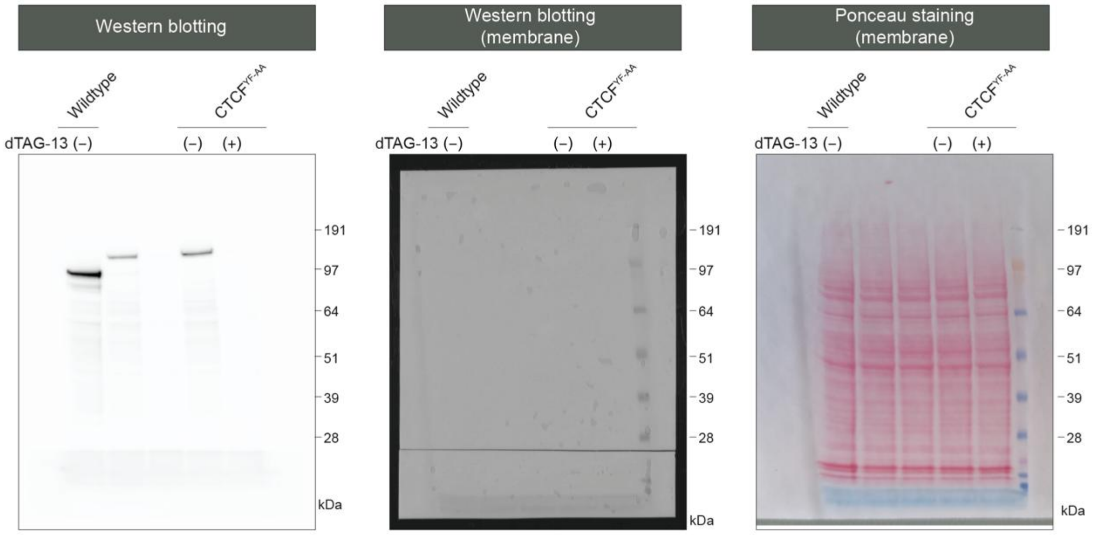

Source image data1. Image Source Data of Extended Data Fig. 7a

Full scan images of immunoblot and ponceau stained membranes of extended data fig. 7a.
